# Gastric tube placement through the drainage channel of second-generation supraglottic airway devices: a systematic review with evidence mapping

**DOI:** 10.3389/fsurg.2026.1867470

**Published:** 2026-06-09

**Authors:** Xinrui Yin, Shijia Du

**Affiliations:** 1 Department of Anesthesiology, Aerospace Center Hospital, Beijing, China; 2 Department of VIP Dental Service, Peking University Stomatological Hospital, Beijing, China

**Keywords:** drainage channel, evidence mapping, gastric tube, general anaesthesia, supraglottic airway, systematic review

## Abstract

**Background:**

Second-generation supraglottic airway (SGA) devices incorporate a dedicated gastric drainage channel that permits placement of a gastric tube (GT), but whether GT placement should be used routinely during general anaesthesia remains uncertain. We conducted a systematic review with evidence mapping to characterize the available evidence and its clinical interpretation across scenarios.

**Methods:**

MEDLINE, Embase, CENTRAL, Web of Science, and Scopus were searched from inception to 18 April 2026 (PROSPERO: CRD420261368854). A prespecified three-tier framework separated direct comparative studies of GT placement versus non-placement (Tier 1) from indirect clinical evidence (Tier 2) and complementary mechanistic or practice-based evidence (Tier 3). Risk of bias was appraised using design-appropriate tools, and GRADE was applied to five prespecified clinical outcomes. Narrative synthesis followed SWiM guidance, and an interpretive scenario-based framework was derived from the synthesis.

**Results:**

Thirty-six studies were included: 2 Tier 1, 33 Tier 2, and 1 Tier 3 study. Across the 35 clinical studies, 4,469 adults contributed data. Tier 1 evidence was limited: Hell et al. reported gastric insufflation in 10.9% of patients with a GT versus 2.7% without (*P* = 0.009), whereas Freisburger and Goldmann found no material change in oropharyngeal leak pressure with a GT *in situ* (*P* = 0.6). Tier 2 evidence did not identify a clear signal of clinically important gastric insufflation, regurgitation, or pulmonary aspiration attributable to GT placement, but outcome definitions and detection methods were heterogeneous. GRADE certainty was Low for gastric insufflation and oropharyngeal leak pressure and Very low for regurgitation, pulmonary aspiration, and postoperative nausea and vomiting. Clinical scenarios were grouped into three interpretive categories reflecting indirect evidence signals consistent with GT placement, no evidence supporting routine GT placement, or insufficient or context-limited evidence.

**Conclusion:**

Current evidence is most consistent with a scenario-dependent interpretation of GT placement through the drainage channel of second-generation SGAs, rather than routine universal placement or universal avoidance; the available sample sizes were insufficient to exclude clinically important differences in rare safety outcomes. Direct GT-strategy trials are needed in underrepresented scenarios.

**Systematic Review Registration:**

https://www.crd.york.ac.uk/prospero/display_record.php?RecordID=1368854, identifier CRD420261368854.

## 1 Introduction

Second-generation supraglottic airway (SGA) devices have become a routine option for airway management in adults undergoing general anaesthesia (1, 2). A defining feature of these devices, distinguishing them from first-generation laryngeal mask airways, is a dedicated gastric drainage channel that permits placement of a gastric tube (GT) for gastric decompression or for routing of regurgitant contents away from the glottis (3). The LMA ProSeal, LMA Supreme, i-gel, Ambu AuraGain, LMA Protector, and several related devices all incorporate this design. Yet the existence of a drainage channel does not determine whether a GT should be placed; in clinical practice, GT insertion through the drainage channel remains highly variable, and its role in reducing gastric-related perioperative risk is not established (4).

The available evidence on this question is dispersed and uneven. Most randomized trials involving second-generation SGAs have been designed as device-to-device comparisons or against tracheal intubation, rather than as direct comparisons of GT placement versus no GT placement (5–7). GT management is frequently reported as a procedural detail rather than as the principal intervention under study. Direct comparative evidence on GT placement itself is therefore sparse, indirect clinical evidence is broader but heterogeneous in outcome definition and measurement, and several clinically important scenarios (obstetric emergency, prone positioning, laparotomy, and higher-risk adult populations) are addressed only by context-limited bodies of evidence (8–10). These features make the question difficult to answer from any single study or simple pairwise meta-analysis.

We therefore conducted a systematic review with evidence mapping and an interpretive scenario-based framework to characterize the current state of evidence on this clinical question. The review was organized around three methodological elements matched to the observed evidence structure: a prespecified three-tier evidence structure that separated direct comparative studies of GT placement versus non-placement (Tier 1) from indirect clinical studies in which GT management was explicitly described alongside relevant outcomes (Tier 2) and from complementary mechanistic and practice-based evidence (Tier 3); an evidence map summarizing the distribution of available evidence across outcomes and evidence sources; and an interpretive scenario-based framework linking each clinical scenario to its supporting evidence and certainty. Design-appropriate appraisal and certainty assessment were used to support this structure. The objective was not to issue clinical practice recommendations but to characterize the structure and certainty of the available evidence, and to make explicit how its certainty and limits shape interpretation across commonly encountered clinical scenarios.

## 2 Methods

### 2.1 Study design and reporting

This systematic review was conducted and reported in accordance with the PRISMA 2020 statement (11), with the narrative synthesis components additionally reported using the Synthesis Without Meta-analysis (SWiM) guideline (12). The review design was informed by the anticipated heterogeneity of the evidence base, which was expected to include direct comparative studies, indirect clinical evidence, and complementary mechanistic or practice-based studies relevant to gastric tube (GT) placement through second-generation supraglottic airway devices (SGAs). A three-tier evidence framework was prespecified to accommodate this heterogeneity, comprising Tier 1 (direct comparative studies of GT placement versus non-placement through the drainage channel of a second-generation SGA), Tier 2 (indirect clinical studies in which GT management was explicitly described alongside relevant gastric-related outcomes), and Tier 3 (simulation, cadaveric, mechanistic, and practice-survey studies). Different eligibility criteria, risk-of-bias assessment tools, and synthesis strategies were applied to each tier according to its evidentiary role. The review also included an evidence mapping component and derived a scenario-based interpretive framework rather than a recommendatory output. The protocol was prospectively registered with PROSPERO (CRD420261368854; registered 14 April 2026) before formal literature screening commenced and was followed without protocol deviations affecting the primary review question.

### 2.2 Eligibility criteria

Studies were eligible if they enrolled adults (≥ 18 years) undergoing general anaesthesia with a second-generation SGA, operationally defined as any SGA incorporating a dedicated gastric drainage channel (including but not limited to the LMA ProSeal, LMA Supreme, i-gel, Ambu AuraGain, and LMA Protector). No restrictions were applied with respect to surgical specialty, patient position, ventilation mode, or baseline perioperative risk. The intervention of interest was GT insertion through the dedicated drainage channel, including orogastric or nasogastric tubes used for gastric access, decompression, or drainage; both continuous intraoperative placement and intermittent insertion-and-removal strategies were eligible. The primary comparator was no GT insertion, defined as the drainage channel left open or capped without a GT in place.

A prespecified three-tier logic governed eligibility across evidence tiers according to each study's evidentiary role in addressing the review question, rather than by study design alone. Tier 1 eligible designs comprised randomized controlled trials, quasi-randomized comparative trials, and other prospective comparative studies directly evaluating GT insertion versus non-insertion through the drainage channel of a second-generation SGA. Tier 2 eligible designs included randomized trials and observational clinical studies in which GT placement was not the primary intervention of interest but in which the GT management strategy was explicitly described and at least one relevant gastric-related outcome was reported; for Tier 2, studies comparing a second-generation SGA against tracheal intubation or a first-generation SGA were eligible only when the second-generation arm included GT placement, drainage-channel use, or reporting of gastric-related outcomes relevant to the review question. Tier 3 eligible designs included simulation studies, cadaveric studies, mechanistic studies, and cross-sectional practice surveys of anaesthesia providers addressing GT placement, drainage-channel use, or related practice patterns.

Paediatric-only studies, studies using first-generation SGAs without a gastric drainage channel, studies of prehospital or cardiopulmonary resuscitation airway management, studies using SGAs solely as conduits for tracheal intubation without evaluating gastric-related outcomes, case reports, studies with fewer than 10 patients, conference abstracts without extractable original data, and studies in which GT management could not be determined were excluded. No language restrictions were applied at the search stage; non-English reports were translated as needed.

### 2.3 Information sources and search

MEDLINE (via PubMed), Embase (via Ovid), the Cochrane Central Register of Controlled Trials (CENTRAL), Web of Science Core Collection, and Scopus were searched from inception to 18 April 2026. The search strategy combined controlled vocabulary and free-text terms for second-generation SGAs and for GT placement or drainage-channel use, and was developed iteratively with input from a medical librarian. Backward and forward citation tracking of included studies and relevant reviews was performed. Study authors were contacted by email when reports lacked sufficient detail to determine GT management or outcome data, up to two attempts over four weeks. Full search strategies for all databases are provided in Supplementary Appendix 1.

### 2.4 Study selection and data extraction

Two reviewers (XRY and SJD) independently screened records in Rayyan after deduplication in EndNote X20 (13), and extracted data using a structured Microsoft Excel form piloted on five studies spanning all three evidence tiers. Evidence tier assignment was undertaken during full-text review according to each study's primary evidentiary role in addressing the review question. Disagreements were resolved by consensus with reference to the prespecified eligibility criteria and registered protocol. Core extracted variables included bibliographic details, study design, key participant and procedural characteristics, second-generation SGA device type, GT management (insertion, timing, duration, suction strategy), perioperative context, and prespecified outcomes. Additional tier-specific variables were extracted to support cluster-based narrative synthesis and evidence mapping.

### 2.5 Outcomes

The primary outcome was gastric insufflation during airway management with a second-generation SGA, with ultrasonographic change in gastric antral cross-sectional area or derived gastric volume as the preferred operational definition; studies using alternative detection methods (epigastric auscultation, surgeon-rated gastric distension, direct observation of air escape through the drainage channel) were eligible and synthesized separately, reflecting differences in the underlying constructs and measurement properties captured by these methods. Secondary outcomes included regurgitation, pulmonary aspiration, postoperative nausea and vomiting (PONV), oropharyngeal leak pressure (OLP), pharyngolaryngeal morbidity, GT insertion success or ease, and gastric drainage volume.

### 2.6 Risk of bias and methodological appraisal

Methodological quality was appraised using tools selected to match the design and evidentiary role of each evidence tier. For randomized controlled trials in Tier 1 and Tier 2, risk of bias was assessed using the Cochrane Risk of Bias 2 (RoB 2) tool (14), with the RoB 2 extension for crossover designs applied where appropriate; an overall judgment was assigned as low risk of bias, some concerns, or high risk of bias. For non-randomized comparative or before-after interventional clinical studies, risk of bias was assessed using the Risk Of Bias In Non-randomized Studies – of Interventions (ROBINS-I) tool (15). For single-arm prospective cohorts, retrospective audits, or other descriptive clinical designs for which neither RoB 2 nor ROBINS-I was applicable, methodological quality was appraised using the Joanna Briggs Institute (JBI) critical appraisal checklist for case series (16). The Newcastle–Ottawa Scale was prespecified as a potential tool for observational cohort or case-control studies not amenable to ROBINS-I but retaining a concurrent comparator; its application was anticipated only if required by the final set of included studies.

Tier 3 studies were appraised using a prespecified structured checklist, with an AXIS-informed assessment applied to any embedded practice-survey component, and were used only for contextual rather than pooled clinical inference (17).

Two reviewers appraised each study independently, with disagreements resolved by consensus. Risk-of-bias and methodological quality judgments informed the study-level appraisal, the narrative synthesis, and the GRADE judgments.

### 2.7 Data synthesis

The synthesis strategy was prespecified to accommodate heterogeneity across the three evidence tiers and to support derivation of the scenario-based interpretive framework. Structured narrative synthesis in accordance with SWiM principles was prespecified as the primary analytical approach, whereas pairwise meta-analysis was planned only as a conditional component. Clinical effect estimates from Tier 1 and Tier 2 studies were synthesized separately from Tier 3 complementary evidence, which was used to provide contextual information on feasibility, failure modes, and practice patterns but was not combined with Tier 1 or Tier 2 evidence for effect estimation.

Pairwise random-effects meta-analysis using the restricted maximum likelihood estimator with Hartung–Knapp adjustment was planned only when at least two Tier 1 direct-comparison studies reported the same outcome under sufficiently comparable conditions (18). Sufficient clinical and methodological homogeneity was operationalized as meeting three criteria: (i) reporting of the same outcome of interest with comparable measurement methods; (ii) comparable population characteristics with respect to age, surgical setting, and perioperative risk profile; and (iii) comparable airway-management context, including ventilation strategy and positioning. If fewer than two Tier 1 studies met all three criteria for a given outcome, that outcome was not pooled quantitatively and was synthesized narratively with explicit identification of the unmet homogeneity criterion.

Structured narrative synthesis was performed first within each evidence tier and then across tiers, organized by prespecified clinical domains including surgical context, patient position, SGA characteristics, patient risk profile, and ventilation strategy, with the specific strata applied according to data availability. Within each stratum, Tier 1 and Tier 2 findings were summarized separately before cross-reference; when available evidence within a stratum was insufficient for meaningful synthesis, the stratum was identified as an evidence gap rather than interpreted by extrapolation.

An evidence map was used to characterize the distribution of available evidence across outcomes and evidence sources. A scenario-based interpretive framework was then derived from the integrated synthesis to support interpretation of GT placement decisions rather than to issue formal clinical recommendations. For each clinical scenario, the potential benefits and harms of GT placement were considered in light of the direction, consistency, and credibility of Tier 1 and Tier 2 findings, together with contextual information from Tier 3 studies on device feasibility and practice variation. Each scenario was grouped into one of three prespecified interpretive categories: (i) scenarios with indirect evidence signals consistent with GT placement; (ii) scenarios with no evidence supporting routine GT placement; and (iii) scenarios with insufficient or context-limited evidence. Classifications were linked explicitly to the evidence tables and certainty assessments and should be interpreted as summarizing directions supported by the available evidence rather than as clinical practice recommendations.

Narrative conclusions were derived through a structured, prespecified procedure rather than through vote counting. Within each cluster, contributing studies were tabulated by outcome domain together with study design, sample size, direction and magnitude of findings, measurement method, and risk-of-bias judgement. A summary statement for each outcome domain was then formulated based on the consistency of effect direction across studies, the comparability of measurement methods, and the methodological quality of the contributing evidence. When findings conflicted, the source of the discrepancy was examined first — including device pair, measurement method, population characteristics, ventilation strategy, and risk-of-bias profile — before a summary statement was formulated; conflicts traceable to such methodological differences were reported as such rather than reconciled by majority count. Studies assessed at serious risk of bias under ROBINS-I or with methodological limitations under JBI appraisal were not used to strengthen narrative conclusions and were retained only as context-limited evidence. Tier 3 evidence was used only to characterize feasibility, failure modes, and practice patterns, and was not used to support clinical effect conclusions. Quantitative analyses, when performed, were conducted in R (version 4.3 or later) using the meta and metafor packages (19).

### 2.8 Certainty of evidence

The certainty of evidence was assessed using the GRADE framework for five prespecified outcomes of principal clinical importance: gastric insufflation (primary), regurgitation, pulmonary aspiration, postoperative nausea and vomiting, and oropharyngeal leak pressure (20). GRADE was applied only to Tier 1 and Tier 2 clinical evidence; Tier 3 evidence was not included because it was not intended to contribute pooled clinical effect estimates. For each outcome, the body of evidence was evaluated across the standard GRADE domains of risk of bias, inconsistency, indirectness, imprecision, and publication bias. Two reviewers performed GRADE assessments independently, with disagreements resolved by consensus.

## 3 Results

### 3.1 Study selection

**Figure 1:**
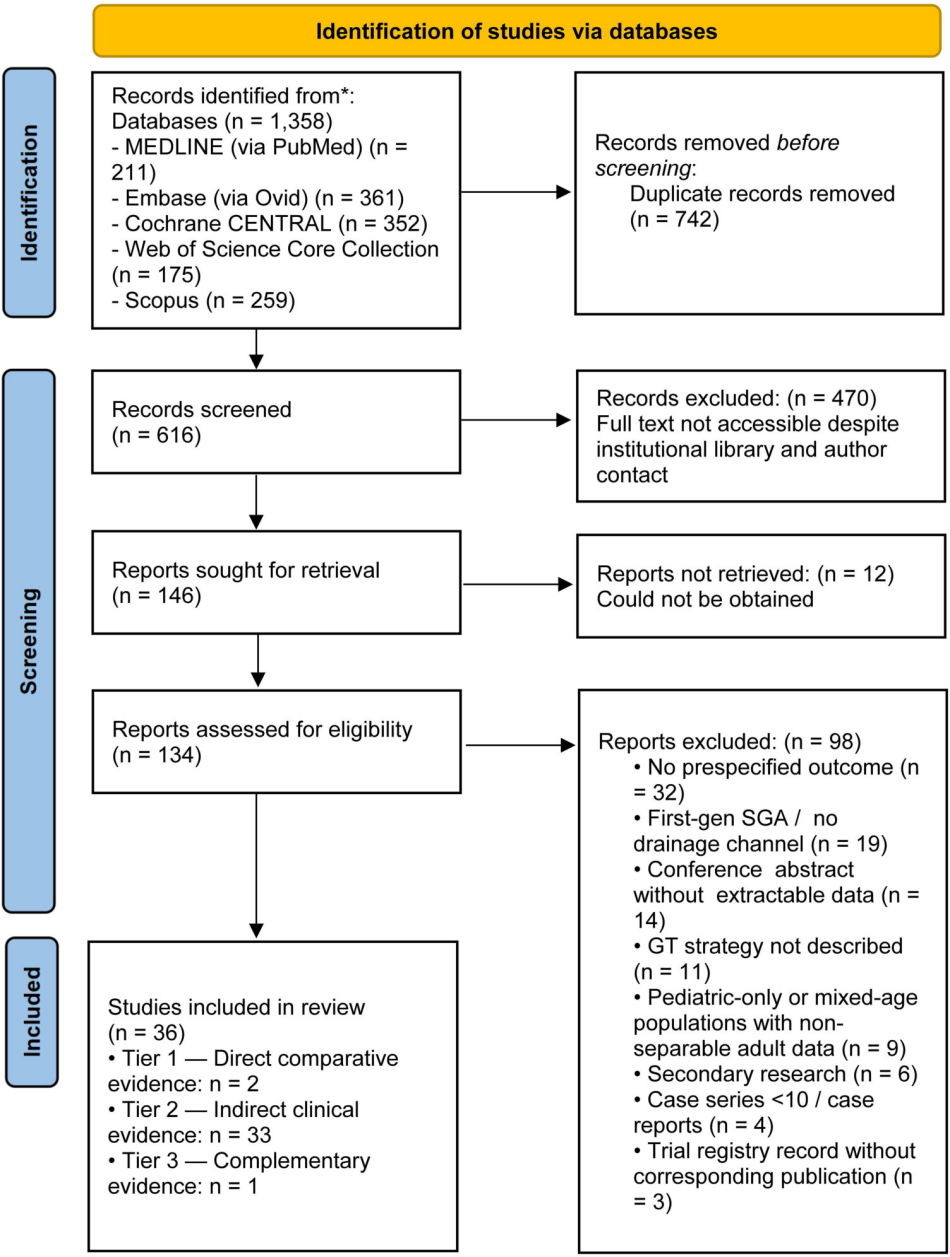
PRISMA 2020 flow diagram of study selection process. Records were identified from five bibliographic databases (MEDLINE, Embase, Cochrane CENTRAL, Web of Science Core Collection, and Scopus) searched from database inception to April 18, 2026. Databases searched: MEDLINE via PubMed (*n* = 211), Embase via Ovid (*n* = 361), Cochrane CENTRAL (*n* = 352), Web of Science Core Collection (*n* = 175), Scopus (*n* = 259). Abbreviations: SGA, supraglottic airway; GT, gastric tube.

The database search identified 1,358 records. After deduplication, 616 unique records underwent title-and-abstract screening. Full-text eligibility assessment was performed for 134 of 146 reports (12 could not be retrieved despite repeated attempts); inter-reviewer agreement at the full-text stage was almost perfect (Cohen's *κ* = 0.86). Ninety-eight reports were excluded at the full-text stage; reasons are detailed in the PRISMA flow diagram (Figure 1). A total of 36 studies met the eligibility criteria: 2 Tier 1 studies, 33 Tier 2 studies, and 1 Tier 3 study.

### 3.2 Study characteristics

The 36 included studies were published between 2000 and 2026, with 19 studies (53%) published since 2015. The included studies were conducted across a broad international range of settings, with notable contributions from India and China, alongside several European centres; one study was an international multicentre trial. Across the 35 clinical studies in Tiers 1 and 2, 4,469 adult patients contributed data.

Study designs were predominantly randomized, comprising 26 parallel-group randomized controlled trials and 2 crossover RCTs. The remaining studies comprised four prospective single-arm or observational cohort studies, one before–after clinical study, one non-randomized comparative clinical study, one retrospective audit, and one prospective manikin-based study with an embedded practice survey. Patients were predominantly adults undergoing elective general anaesthesia in tertiary or academic hospital settings. Some Tier 2 studies used endotracheal intubation or the first-generation LMA Classic as the comparator; these studies were eligible under the prespecified criteria because the second-generation SGA arm included gastric tube placement or reported gastric-related outcomes.

The second-generation SGAs examined encompassed the full spectrum of designs available during the study period. The LMA ProSeal was the most frequently investigated device, followed by the LMA Supreme, the i-gel, the Ambu AuraGain, and the LMA Protector; less commonly studied devices included the Laryngeal Tube Suction and LTS II, the Guardian LMA, and the SaCoVLM video laryngeal mask. Surgical contexts spanned elective general anaesthesia, laparoscopic surgery (gynaecological, urological, and cholecystectomy), laparotomy, ambulatory tubal ligation, caesarean delivery, and ophthalmic and orthopaedic surgery. Patients were predominantly managed in the supine position, with specific subgroups of studies examining the lateral decubitus position for laparoscopic urological surgery, the prone position, and Trendelenburg positioning during gynaecological laparoscopy. A summary of the evidence base by evidence tier and Tier 2 cluster is presented in Table 1, and detailed per-study characteristics are provided in Supplementary Table S1 (Tier 1), 2 (Tier 2, with Cluster A/B/C assignment), and 3 (Tier 3).

**Table 1:** Summary characteristics of the evidence base, by evidence tier and Tier 2 cluster.

Evidence tier/cluster	Studies, n	Patients, n	Study designs	Most frequent devices	Principal surgical contexts	Principal outcomes reported
Tier 1 — Direct comparative evidence	2	250	Crossover RCT (1); before–after clinical study (1)	LMA ProSeal; i-gel	Elective general anaesthesia; elective ophthalmic surgery	Gastric insufflation; oropharyngeal leak pressure
Tier 2 Cluster A — GT and drainage-channel performance	20	2,313	Parallel RCT (18); crossover RCT (1); prospective observational (1)	LMA ProSeal; LMA Supreme; i-gel; Ambu AuraGain; LMA Protector	Elective general anaesthesia; laparoscopic surgery; gynaecological laparoscopy	GT insertion success/time/ease; oropharyngeal leak pressure; drainage function
Tier 2 Cluster B — Gastric-related safety outcomes	6	677	Parallel RCT (5); prospective observational cohort (1)	LMA ProSeal; LMA Supreme; i-gel; Ambu AuraGain; LMA Protector	Laparoscopic surgery; gynaecological laparoscopy; geriatric elective surgery	Gastric insufflation; regurgitation; pulmonary aspiration; postoperative nausea and vomiting
Tier 2 Cluster C — Special clinical scenarios	7	1,229	Parallel RCT (3); non-randomized comparative (1); prospective single-arm series (2); retrospective audit (1)	LMA ProSeal; LMA Supreme; i-gel; Ambu AuraGain; SaCoVLM	Emergency caesarean delivery; postpartum tubal ligation; laparotomy; prone position; lateral retroperitoneal laparoscopy	GT insertion success; oropharyngeal leak pressure; regurgitation; pulmonary aspiration
Tier 3 — Complementary evidence	1	32 anaesthesiologists	Manikin study with embedded practice survey	i-gel; LMA Supreme; LMA ProSeal; Ambu AuraGain	Not applicable (manikin)	GT insertion time and ease; practice patterns for routine GT use
Total (clinical studies, Tiers 1 + 2)	35	4,469	—	—	—	—

GT, gastric tube; LMA, laryngeal mask airway; RCT, randomized controlled trial; SGA, supraglottic airway.

Sample sizes refer to the analysed adult study population in Tiers 1 and 2; for Tier 3 (4), n refers to the number of anaesthesiologists evaluated in the embedded practice survey. Detailed per-study characteristics for all 36 included studies, including first author, year, country, ASA status, sample size, surgical setting, airway device, GT/drainage strategy, and key findings, are provided in Supplementary Table S1 (Tier 1), Supplementary Table S2 (Tier 2, with Cluster A/B/C assignment), and Supplementary Table S3 (Tier 3).

### 3.3 Risk of bias

All 28 randomized trials were judged at “Some concerns” under Cochrane RoB 2; no trial reached overall low risk, and no trial was judged at high risk. The most frequent contributors were unclear allocation concealment, absence of an accessible prespecified analysis plan, and insufficiently described blinding of outcome assessors. The before–after study (21) was judged at moderate risk of bias under ROBINS-I. The non-randomized comparative study (22) was judged at serious risk of bias under ROBINS-I, reflecting clinician-selected allocation and incomplete recruitment-flow reporting. The five single-arm clinical series (23–27) were judged to have moderate JBI limitations. The Tier 3 study (4) had moderate methodological limitations, reflecting simulation-setting constraints and single-institution sampling. Inter-reviewer agreement for domain-level judgements was high, with disagreements resolved by consensus. Domain-level risk-of-bias judgements for the 28 randomized trials (RoB 2) and the 2 non-randomized interventional studies (ROBINS-I) are visualized in Supplementary Figure S1 (Panels A and B, respectively). Study-level appraisal details for all included studies, including the five single-arm series (JBI) and the Tier 3 study, are provided in Supplementary Table S2 and Supplementary Appendices 2A, 2B, and 3.

### 3.4 Tier 1 findings

Two studies provided direct comparative evidence on GT placement through the drainage channel. Meta-analysis was not performed because the two studies reported different primary outcomes under different ventilation conditions, failing the prespecified homogeneity criteria. One study addressed gastric insufflation, and the other addressed oropharyngeal leak pressure. In a randomized crossover trial, Hell et al. examined 152 adults (147 analysed) (28). During an incremental airway pressure trial, gastric insufflation was detected in 16/147 patients (10.9%) with a GT inserted versus 4/147 (2.7%) without a GT (*p* = 0.009), measured by real-time ultrasonographic antral imaging. In a before–after clinical study of 98 adults undergoing elective ophthalmic surgery with the LMA ProSeal, Freisburger and Goldmann (21) reported no material change in airway leak pressure after GT placement (25 ± 6.3 cmH₂O without GT vs 25 ± 6.7 cmH₂O with GT; *P* = 0.6). Taken together, Tier 1 evidence permitted two narrow direct observations: GT placement through the drainage channel increased gastric insufflation during positive-pressure ventilation, and GT placement did not measurably alter oropharyngeal leak pressure. Neither study reached an overall low risk of bias, and each outcome was informed by a single direct-comparison study, precluding any judgement of reproducibility.

### 3.5 Tier 2 findings

The 33 Tier 2 studies were organized into three prespecified thematic clusters reflecting their principal research questions; study-level data are provided in Supplementary Table S2 (29–43).

#### 3.5.1 Cluster A —GT and drainage-channel performance (*n* = 20; 2,313 patients)

Gastric tube insertion through the drainage channel succeeded in the large majority of patients across included studies, typically on the first attempt, although the relative performance between devices varied by device pair. In head-to-head randomized comparisons, the LMA Supreme and the Ambu AuraGain were more often associated with higher first-attempt insertion success, faster insertion times, and better operator-rated ease than the i-gel and the LMA ProSeal; the LMA Protector showed a recurring pattern of higher oropharyngeal leak pressure accompanied by more difficult gastric tube placement than comparator devices; and no single device was uniformly superior across all comparisons.

Representative studies illustrate this pattern. Where the LMA Supreme and the Ambu AuraGain were compared against the i-gel or the LMA ProSeal, gastric tube insertion was both faster and more often successful on the first attempt with the Supreme or AuraGain arm (6, 44); in one direct comparison between the Ambu AuraGain and the LMA Supreme, gastric tube insertion succeeded in 49/49 patients (100%) with the AuraGain versus 40/44 (90.9%) with the LMA Supreme (*P* = 0.046), with similar drainage-tube aspirate volumes between devices (15.3 ± 16.3 mL versus 16.5 ± 18.7 mL; *P* = 0.763) (5). Comparisons involving the LMA Protector illustrated a consistent seal–ease trade-off: against the Ambu AuraGain, first-pass gastric tube insertion was more often successful with the AuraGain and accompanied by shorter insertion times (45); against the i-gel during laparoscopic surgery, gastric tube insertion was slower and rated as more difficult with the Protector, although the Protector's airway seal was higher (46). In a body-weight-adjusted subgroup analysis, selecting the LMA ProSeal size by ideal rather than actual body weight improved gastric tube insertion success in overweight and obese adults (47).

Taken together, and consistent with the underlying study-level data in Supplementary Table S2, gastric tube insertion through the drainage channel was reported as feasible across all second-generation SGAs in the included settings. Comparative performance was device-dependent, with the LMA Supreme and the Ambu AuraGain more often favoured on insertion metrics and the LMA Protector exemplifying a seal–ease trade-off replicated across multiple comparator devices. Absolute between-device differences were modest in most studies, and no device emerged as uniformly superior across outcomes. These observations describe device-level performance characteristics and do not directly inform whether GT placement should be undertaken.

#### 3.5.2 Cluster B — gastric-related safety outcomes (*n* = 6; 677 patients)

Five randomized trials and one prospective multicentre observational study contributed evidence on gastric insufflation, regurgitation, pulmonary aspiration, and postoperative morbidity. Across this small and methodologically heterogeneous cluster, the available evidence did not identify a clear signal of clinically important gastric insufflation, regurgitation, or pulmonary aspiration attributable to GT placement. Gastric insufflation rates differed by detection method and device pair; in a trial focused on geriatric patients, epigastric-auscultation–detected insufflation occurred in 0/19 i-gel patients versus 5/16 LMA Supreme patients (0% vs 31.3%; *P* = 0.013) at the time of OLP measurement (In 2019) (48). Ultrasonographic quantification favoured the Ambu AuraGain over the LMA ProSeal, with smaller gastric insufflation volumes; postoperative adverse-event reporting also numerically favoured the AuraGain, although detailed comparative interpretation remained limited. A prospective multicentre observational cohort of 300 adults with the LMA Protector documented reflux content in the drainage channel in 5/300 patients (1.67%) without clinical evidence of aspiration (26). No cases of clinically confirmed pulmonary aspiration were reported across the six Cluster B studies. However, the contributing sample sizes were insufficient to exclude clinically important differences in such rare events, and the absence of observed events should not be interpreted as evidence of safety.

#### 3.5.3 Cluster C — special clinical scenarios (*n* = 7; 1,229 patients)

Seven Tier 2 studies contributed indirect evidence on gastric tube placement through the drainage channel of second-generation SGAs in clinical contexts that lay outside the routine elective populations examined in Clusters A and B. Across these studies, GT placement through the drainage channel was reported as feasible in obstetric, abdominal, and non-supine surgical contexts, with generally high device-insertion and gastric-tube-placement success rates in the populations studied; findings are summarized by scenario subgroup: obstetric and gynaecological contexts, abdominal surgery, and non-supine positioning.

In the obstetric and gynaecological subgroup, a prospective single-arm series of 584 parturients undergoing category 2 or 3 emergency caesarean delivery with the LMA Supreme reported first-attempt device insertion in 574/584 patients (98.3%), overall insertion success in 584/584, and first-attempt orogastric tube placement through the drain tube in 584/584, with no clinical evidence of regurgitation or aspiration (25). An earlier prospective single-arm series of 90 fasted women undergoing postpartum tubal ligation with the LMA ProSeal similarly reported successful device insertion in all 90 patients and gastric tube placement in 90/90, with no suspected regurgitation or aspiration events (24). In a randomized comparison between the LMA Supreme and the i-gel during ambulatory laparoscopic female sterilization, time to gastric tube insertion was shorter with the LMA Supreme (7.9 ± 1.9 versus 14.8 ± 7.7 s; *P* < 0.001), and gastric tube placement was rated as easy in 35/35 LMA Supreme patients (100%) versus 27/35 i-gel patients (77.1%; *P* = 0.005); no gross regurgitation was observed in either group (50).

In the abdominal surgery subgroup, a non-randomized comparative study of 65 adults (34 LMA ProSeal, 31 tracheal intubation) undergoing elective gynaecological or general laparotomy reported one regurgitation event through the drainage channel without aspiration, with approximately 5 mL of green fluid and approximately 20 mL of gastric contents suctioned during the episode (22); this study was assessed at serious risk of bias under ROBINS-I, and its comparative effect estimates should be interpreted as illustrative rather than confirmatory. In a three-arm randomized trial comparing the Ambu AuraGain, the i-gel, and tracheal intubation during elective laparoscopic cholecystectomy, device insertion succeeded at first attempt in 38/38 with the AuraGain, 35/35 with the i-gel, and 31/32 with the tracheal tube (*P* = 0.876), and no aspiration complications were reported across the three groups (51).

In the non-supine positioning subgroup, a retrospective audit of 245 healthy adults managed in the prone position with the LMA ProSeal by experienced users reported successful device insertion and gastric tube placement in all 245 patients, with no hypoxia, device displacement, regurgitation, gastric insufflation, or airway reflex activation (27). In a randomized comparison between the SaCoVLM video laryngeal mask and the LMA Supreme during retroperitoneal laparoscopic surgery in the lateral position, gastric tube placement through the drain tube succeeded in 35 of 35 patients in each arm (70 patients total), and oropharyngeal leak pressure was consistently higher with the SaCoVLM than with the LMA Supreme across the six measurement time points (all *P* ≤ 0.008); no reflux aspiration was reported in either group, although blood staining on the device was more frequent with the SaCoVLM than with the LMA Supreme (8/35 versus 1/35; *P* = 0.028) (52).

Taken together, Cluster C provides context-specific evidence on feasibility and reported event patterns of gastric tube placement in obstetric, abdominal, and non-supine surgical populations, acknowledging that the available sample sizes within these scenarios were insufficient to exclude clinically important differences in rare safety events. No clinically confirmed pulmonary aspiration was reported across the cluster, acknowledging the rare-event nature of aspiration and the limited sample sizes of the individual studies; the single regurgitation event reported through the drainage channel occurred without aspiration in the non-randomized laparotomy cohort assessed at serious risk of bias (22). Findings from the single-arm obstetric and prone-position cohorts, assessed as having moderate methodological limitations under JBI case-series appraisal, should be interpreted as descriptive rather than comparative evidence.

### 3.6 Tier 3 findings

One manikin-based study with an embedded practice survey (4) evaluated GT insertion performance across four second-generation SGAs in 32 anaesthesiologists. GT insertion times differed significantly across devices (median 8.9–18.8 s; Friedman *P* < 0.001), with the LMA Supreme and the Ambu AuraGain significantly faster than the i-gel and the LMA ProSeal (*P* < 0.001). The LMA Supreme was identified as the easiest (19/32, 59%) and the LMA ProSeal as the most challenging (15/32, 47%) device for GT insertion. In the embedded survey, 23/32 anaesthesiologists (72%) routinely used the i-gel; of these, 18/23 (78%) did not routinely insert a GT when using the i-gel, and none of the respondents cited the possibility of GT insertion as a contributing factor in routine device selection. Tier 3 evidence provided practice-based context broadly aligned with the device-level patterns observed in Tier 2 Cluster A, but was not combined with Tier 1 or Tier 2 evidence for effect estimation or GRADE certainty ratings. Detailed characteristics of this study are presented in Supplementary Table S3.

### 3.7 Evidence map

**Figure 2:**
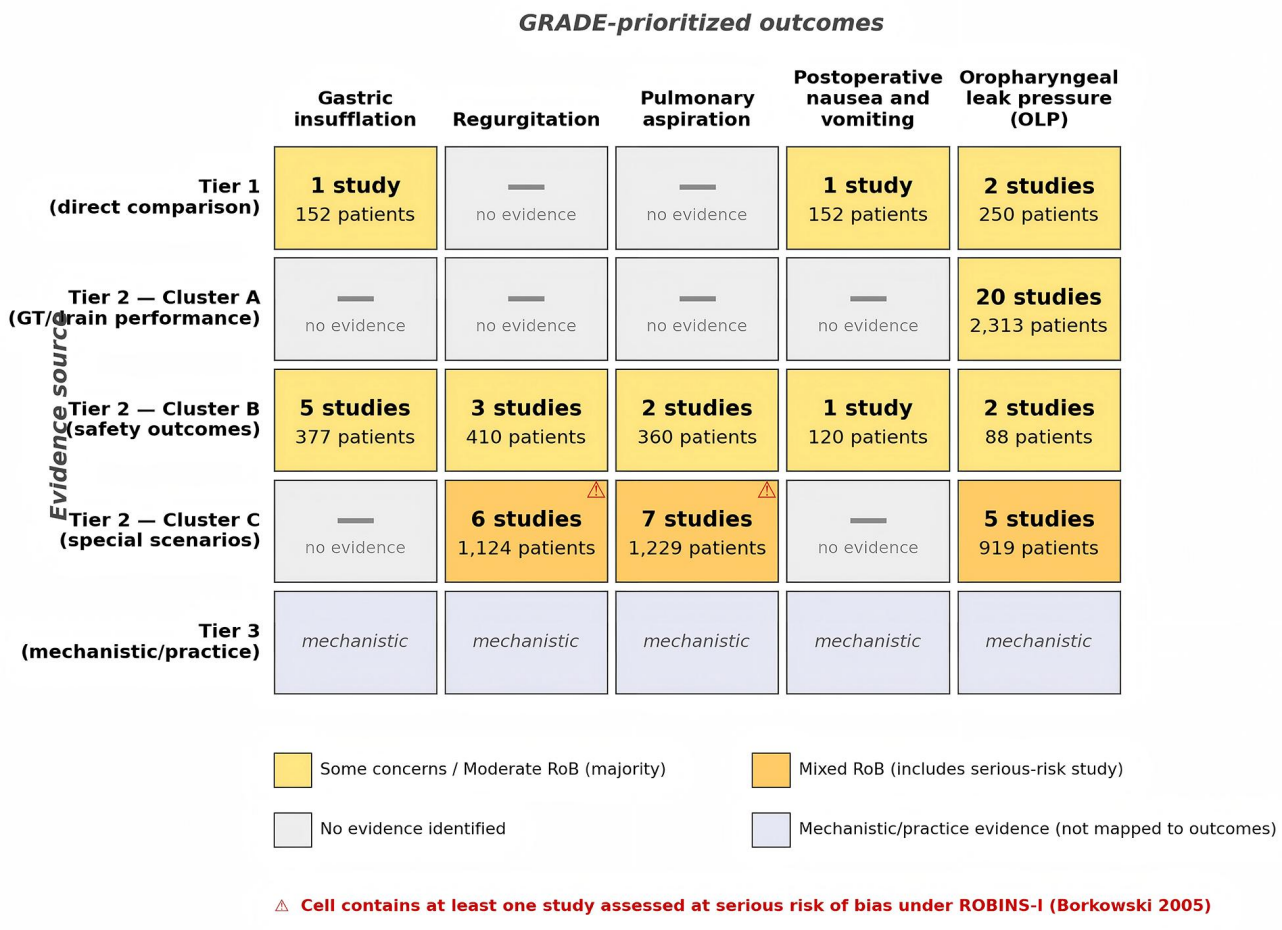
Evidence map of gastric tube placement through the drainage channel of second-generation supraglottic airway devices: evidence distribution across the five gRADE-prioritized outcomes and the five evidence sources mapped in this review. Each cell reports the number of studies and the total number of patients contributing to the corresponding outcome–source pair, with colour indicating the predominant risk-of-bias judgement across the contributing studies (yellow, majority Some concerns under Cochrane RoB 2 or Moderate risk under ROBINS-I or Moderate limitations under JBI case-series appraisal; amber, mixed judgements including at least one study assessed at serious risk of bias). Cells labelled “no evidence” indicate that no included study in that evidence source reported the corresponding outcome. The Tier 3 row is shown in light lavender and labelled “mechanistic” to indicate that mechanistic and practice-based evidence from this tier was not mapped to the clinical outcomes framework. The red warning symbol (⚠) indicates cells containing at least one study assessed at serious risk of bias under ROBINS-I (22). Individual study-level findings are tabulated in Supplementary Tables S1–S3 and Supplementary Table S4. Abbreviations: GT, gastric tube; JBI, Joanna Briggs Institute; OLP, oropharyngeal leak pressure; RoB 2, Cochrane risk of bias 2 tool; ROBINS-I, risk of bias in non-randomized studies – of interventions.

The distribution of evidence across the five GRADE-prioritized outcomes and five evidence sources is summarized in Figure 2. Oropharyngeal leak pressure was the most extensively reported outcome across the evidence base, spanning Tier 1 and all three Tier 2 clusters, with 29 contributing studies and 3,570 patients. Direct Tier 1 evidence was limited overall: gastric insufflation and postoperative nausea and vomiting were each informed by a single study (28), whereas OLP was informed by two studies (21, 28); direct Tier 1 evidence was absent for regurgitation and pulmonary aspiration, both of which were informed entirely by indirect Tier 2 evidence.

The three Tier 2 clusters contributed to different parts of the outcome matrix. Cluster A contributed extensive evidence on oropharyngeal leak pressure but did not contribute directly to any of the four safety outcomes, reflecting the focus of these studies on gastric tube and drainage-channel performance rather than gastric-related safety. Cluster B contributed evidence across all five outcomes, though study numbers and patient samples for individual cells remained modest. Cluster C contributed the largest number of studies for both regurgitation and pulmonary aspiration; these cells included one non-randomized comparative study assessed at serious risk of bias under ROBINS-I (22), which is flagged in the evidence map. Tier 3 contributed mechanistic and practice-based evidence on gastric tube insertion performance but did not contribute outcome-level data directly mappable to the five GRADE-prioritized outcomes.

Two evidence gaps were particularly notable. First, no direct Tier 1 evidence was identified for regurgitation or pulmonary aspiration, and the Tier 2 base for these two outcomes combined randomized trials, a non-randomized comparative study at serious risk of bias, and single-arm observational series. Second, postoperative nausea and vomiting was addressed by only two studies in the entire evidence base (Hell (28) and Gunasekaran (49); combined sample size 272 patients); across the evidence map, no cell was supported by studies judged at overall low risk of bias. This pattern of outcome-by-source gaps informed the scenario-based interpretive framework.

### 3.8 Certainty of evidence (GRADE)

The GRADE assessments, summarized in Table 2, were based on the full contributing Tier 1 and Tier 2 clinical evidence for each outcome. Certainty was rated Low (⊕⊕○○) for two outcomes: gastric insufflation (6 studies, 529 patients) and oropharyngeal leak pressure (29 studies, 3,570 patients). Certainty was rated Very low (⊕○○○) for three outcomes: regurgitation (9 studies, 1,534 patients), pulmonary aspiration (9 studies, 1,589 patients), and postoperative nausea and vomiting (2 studies, 272 patients). No outcome was judged to provide high- or moderate-certainty evidence. Domain-level downgrading is summarized in Table 2, and reflected the absence of low-risk-of-bias evidence, the predominance of indirect device-comparison trials over direct GT-placement trials, the rarity of the safety events assessed, and — for regurgitation and pulmonary aspiration — a mixed evidence base that included single-arm observational series and one non-randomized comparative study assessed at serious risk of bias (22).

**Table 2:** Summary of findings: GRADE certainty of evidence for the five prespecified outcomes in this systematic review of gastric tube placement through the drainage channel of second-generation supraglottic airway devices in adults undergoing general anaesthesia.

Outcome	Studies contributing (n)	Patients (N)	Risk of bias	Inconsistency	Indirectness	Imprecision	Publication bias	Initial certainty	Downgrades	Final certainty	Summary of findings
Gastric insufflation^d^	6	529	Serious (–1)	Not serious	Not serious	Serious (–1)	Not detected	High (⊕⊕⊕⊕)	–2	Low (⊕⊕○○)	Gastric tube placement through the drainage channel may increase the occurrence of gastric insufflation during positive-pressure ventilation; direction of effect supported by Tier 1 direct evidence (4), with Tier 2 evidence reporting generally low rates of clinically important insufflation under varied detection methods
Regurgitation	9	1,534	Very serious (–2)	Not additionally downgraded^a^	Serious (–1)	Serious (–1)	Not detected	High (⊕⊕⊕⊕)	–4	Very low (⊕○○○)	The available evidence does not identify a clear signal of clinically important regurgitation attributable to gastric tube placement through the drainage channel; observed events were rare across the evidence base
Pulmonary aspiration	9	1,589	Very serious (–2)	Not additionally downgraded^a^	Serious (–1)	Very serious (–2)	Not detected	High (⊕⊕⊕⊕)	–5 (floored)^b^	Very low (⊕○○○)	No case of clinically confirmed pulmonary aspiration was reported across the contributing patients; the rarity of events and absence of direct Tier 1 evidence preclude precise risk estimation
Postoperative nausea and vomiting	2	272	Serious (–1)	Not evaluable^c^	Serious (–1)	Very serious (–2)	Not detected	High (⊕⊕⊕⊕)	–4	Very low (⊕○○○)	Limited data; no clear signal of increased postoperative nausea and vomiting attributable to gastric tube placement through the drainage channel
Oropharyngeal leak pressure	29	3,570	Serious (–1)	Not serious	Serious (–1)	Not serious	Not detected	High (⊕⊕⊕⊕)	–2	Low (⊕⊕○○)	Gastric tube placement through the drainage channel does not appear to alter oropharyngeal leak pressure; supported by Tier 1 direct within-patient evidence (21) and consistent with Tier 2 device-comparison findings

GRADE, grading of recommendations assessment, development and evaluation.

GRADE certainty symbols: ⊕⊕⊕⊕ High; ⊕⊕⊕○ Moderate; ⊕⊕○○ Low; ⊕○○○ Very low.

Initial certainty started from High on the basis of the randomized evidence contributing to each outcome; where outcomes were informed by a mixed body of randomized and non-randomized evidence, the composition of the contributing evidence base was reflected in the downgrading decisions. Individual study-level findings and quantitative effect estimates are tabulated in Supplementary Supplementary Table S2; study-level risk of bias is detailed in Supplementary Figure S1 and Supplementary Table S4. Publication bias was assessed qualitatively across the evidence base; formal funnel-plot analysis was not feasible because meta-analysis was not performed.

a Inconsistency was difficult to assess separately for outcomes with very rare events and heterogeneous study designs, and no additional downgrade was applied beyond the risk-of-bias and indirectness considerations already captured.

b For pulmonary aspiration, cumulative downgrading exceeded four levels; in accordance with GRADE convention, the final certainty was floored at Very low.

c Inconsistency was not evaluable because only two studies contributed data, precluding meaningful assessment of between-study heterogeneity.

d Detection methods for gastric insufflation varied across studies, including ultrasonographic antral assessment, epigastric auscultation, surgeon-rated distension, and direct observation of air escape through the drainage channel. These methods capture overlapping but non-equivalent constructs with different sensitivity, specificity, and clinical interpretation. This heterogeneity was considered during GRADE assessment, but no additional inconsistency- or indirectness-domain downgrading was applied on this basis.

### 3.9 Scenario-based interpretive framework

The scenario-based interpretive framework is presented in Table 3. Clinical scenarios were grouped into three prespecified interpretive categories according to the direction and certainty of the available evidence, while preserving the distinction between direct comparative evidence, indirect supportive evidence, and contextual evidence. Category (i) comprised scenarios with indirect evidence signals consistent with GT placement, including intra-abdominal laparoscopic surgery requiring gastric decompression, clinical situations requiring continuous gastric drainage, and selected elective non-laparoscopic situations in which gastric drainage is clinically indicated. Category (ii) comprised scenarios with no evidence supporting routine GT placement, including short-duration elective surgery in fasted, low-risk adults and elective settings in which the available indirect evidence showed no clear advantage of routine GT insertion. Category (iii) comprised scenarios with insufficient or context-limited evidence, including emergency obstetric surgery, prone-position surgery, laparotomy, and populations not directly addressed by the evidence base, such as morbidly obese adults, paediatric patients, and patients with full stomach. These categories are intended to summarize evidence signals and uncertainty, not to recommend or discourage GT placement in individual patients. Several classifications remain based predominantly on indirect, single-arm, or otherwise context-limited evidence, and should be interpreted alongside the certainty ratings and limitations summarized in Tables 2, 4.

**Table 3:** Scenario-based interpretive framework for evidence signals and uncertainty regarding gastric tube placement through the drainage channel of second-generation supraglottic airway devices in adults undergoing general anaesthesia.

Interpretive category	Clinical scenario	Main contributing evidence	Certainty of relevant outcome(s)	Interpretive summary of evidence signal and uncertainty
Indirect evidence signals consistent with GT placement	Intra-abdominal laparoscopic surgery requiring gastric decompression	Cluster A (LMA Supreme/AuraGain head-to-head trials); Cluster C laparoscopic studies	Low (gastric insufflation, OLP); Very low (regurgitation, aspiration)	Available indirect evidence was consistent with technical feasibility of GT placement and gastric-content evacuation in laparoscopic settings where decompression was clinically relevant, without a clear observed signal of increased clinically confirmed regurgitation or compromised airway seal. However, evidence for rare safety outcomes remained Very low certainty.
Indirect evidence signals consistent with GT placement	Clinical situations requiring continuous gastric drainage	Tier 1 OLP study (21); Tier 2 ProSeal/Supreme drain-channel studies	Low (OLP); Very low (regurgitation, aspiration)	Available evidence was consistent with feasibility of GT placement when continuous gastric drainage was part of the airway-management strategy, and Tier 1 evidence did not suggest an appreciable reduction in airway seal with a GT in situ. Evidence for effects on regurgitation or aspiration remained Very low certainty.
Indirect evidence signals consistent with GT placement	Selected elective non-laparoscopic situations in which gastric drainage is clinically indicated	Tier 1 (21); Cluster A elective surgery studies	Low (OLP); Very low (regurgitation, aspiration)	Available evidence was consistent with preserved airway seal when a GT was present and with procedural feasibility in selected elective settings. Any inference that GT placement reduces regurgitation or pulmonary aspiration remains unsupported by direct evidence and is limited by Very low certainty for rare safety outcomes.
No evidence supporting routine GT placement	Short-duration elective surgery in fasted, low-risk adults	Cluster A elective studies; Tier 1 (28)	Low (gastric insufflation, OLP)	Available evidence did not identify a clear advantage signal supporting routine GT placement in fasted, low-risk, short-duration elective surgery. Tier 1 evidence also raised a context-specific signal of increased ultrasonographic gastric insufflation during pressure testing with a GT in situ.
No evidence supporting routine GT placement	Elective settings in which device-specific differences in GT insertion performance have been reported	Cluster A device-comparison RCTs	Low (OLP); Very low (other outcomes)	Available indirect evidence reported device-specific differences in GT insertion performance, with certain devices associated with slower or less successful insertion in head-to-head comparisons. These observations describe device-level performance characteristics and do not directly inform whether GT placement should be undertaken.
Insufficient or context-limited evidence	Emergency obstetric surgery	Single-arm cohort ((25), *n* = 584)	Very low	GT placement was reported as feasible in a large single-arm obstetric cohort, with no clinically confirmed aspiration events reported. However, the absence of randomized or comparative evidence, the acuity of the population, and the rarity of aspiration events preclude a firm scenario-specific conclusion.
Insufficient or context-limited evidence	Prone-position surgery	Retrospective audit ((27), *n* = 245)	Very low	GT placement was reported as feasible in a single specialist-centre audit conducted by experienced users. The absence of randomized evidence, the operator-dependent nature of the findings, and limited generalizability preclude a firm scenario-specific conclusion.
Insufficient or context-limited evidence	Laparotomy	One non-randomized comparative study ((22), serious RoB)	Very low	The only identified evidence was assessed at serious risk of bias and included one regurgitation event vented through the drainage channel without aspiration. No firm scenario-specific conclusion can be drawn, and the evidence summarized here does not support a scenario-specific interpretation for laparotomy.
Insufficient or context-limited evidence	Populations not directly addressed by the evidence base (morbidly obese adults, paediatric patients, patients with full stomach)	None	Not applicable (no contributing evidence)	No scenario-specific conclusion can be drawn from the current evidence base. These populations remain evidence gaps, and extrapolation from the studies summarized in this framework is not supported.

Category classifications were derived according to the prespecified synthesis approach used in this review and summarize evidence signals and uncertainty rather than clinical practice recommendations. Main contributing evidence refers to the studies most directly relevant to each scenario, which may provide direct comparative, indirect supportive, or contextual evidence depending on the tier; full study-level findings are provided in the narrative synthesis and in Supplementary Tables S1–S3. Certainty ratings are reproduced from Table 2 and reflect the GRADE-assessed outcomes most directly relevant to each scenario; Tier 3 evidence was not included in GRADE assessments. Interpretive summaries should not be read as recommendations for or against GT placement in individual patients; clinical management remains within the scope of formal airway-management guidelines, institutional protocols, and individual clinical judgement. Evidence in this framework was derived from adults undergoing general anaesthesia with second-generation SGAs, and extrapolation beyond these populations, devices, and clinical contexts is not supported.

**Table 4:** Key evidence gaps and interpretive limitations identified in this systematic review.

Evidence domain/scenario	Current evidence	Main limitation	Implication for interpretation	Priority for future research
Direct GT vs no-GT evidence	2 Tier 1 studies, each informing a single outcome (gastric insufflation; OLP)	Neither study reached overall low risk of bias; pooled analysis precluded by outcome non-overlap	Direct effect of GT strategy remains poorly characterized	High
Gastric insufflation measurement	Reported by ultrasonographic antral assessment, epigastric auscultation, surgeon-rated distension, and direct observation of air escape	Detection methods capture overlapping but non-equivalent constructs and were not interchangeable for synthesis	Effect-direction interpretation must be method-specific	High — adoption of standardized ultrasonographic protocols
Regurgitation	9 Tier 2 studies, no direct Tier 1 evidence	Inconsistent operational definitions; rare-event detection limited by sample size	Absence of clear signal cannot be equated with evidence of safety	Medium — consensus operational definition required
Pulmonary aspiration	9 Tier 2 studies, no clinically confirmed events	Sample size insufficient to exclude clinically meaningful differences in rare events	Reported zero events do not constitute evidence of safety	Medium to high — multicentre prospective registries or large pragmatic trials
Postoperative nausea and vomiting	2 studies, 272 patients	Insufficient to support meaningful inference; Very low GRADE certainty	No interpretable signal at present	Medium
Emergency obstetric surgery	One large single-arm cohort (*n* = 584), no comparative evidence	Acuity, rare-event nature of aspiration, absence of randomized evidence	No firm scenario-specific interpretation possible	High
Prone positioning	One single-centre retrospective audit by experienced users	Operator-dependent feasibility, no comparative evidence	Generalizability limited	High
Laparotomy	One non-randomized comparative study at serious risk of bias under ROBINS-I	Single seriously biased study cannot support scenario-specific interpretation	Evidence base insufficient to support scenario-specific interpretation	High
Underrepresented populations (morbidly obese, paediatric, full stomach)	No direct evidence	Complete evidence gap	Extrapolation from current evidence not supported	High — dedicated cohort or trial designs

This table summarizes evidence gaps and interpretive limitations identified across the synthesis and is intended to support transparent interpretation of the framework presented in Table 3.

## 4 Discussion

The overall picture emerging from this review is that the available evidence does not support a uniform routine-use or routine-avoidance strategy for gastric tube (GT) placement through the drainage channel of second-generation supraglottic airway devices, and instead points to a scenario-dependent interpretation anchored in the structure and certainty of the underlying evidence. Direct comparative evidence was limited to two Tier 1 studies, each informing a single outcome and neither reaching overall low risk of bias. In a randomized crossover trial, Hell et al. (28) reported that GT placement was associated with a within-patient increase in ultrasonographically detected gastric insufflation during an incremental airway pressure trial. In a before–after clinical study, Freisburger and Goldmann (21) reported that oropharyngeal leak pressure (OLP) was preserved with a GT in situ. Thirty-three Tier 2 studies contributed indirect clinical evidence across three thematic clusters (GT and drainage-channel performance, gastric-related safety outcomes, and special clinical scenarios) and did not identify a clear signal of clinically important gastric insufflation, regurgitation, or pulmonary aspiration attributable to GT placement. However, this absence of a clear signal cannot be equated with evidence of safety, given the rarity of these events and the limited sample sizes contributing to each outcome. No single device was uniformly superior across comparisons, and detection methods for gastric insufflation varied substantially across studies. One Tier 3 study provided mechanistic and practice-based context on GT insertion performance and real-world practice variation. GRADE certainty was Low for gastric insufflation and OLP and Very low for regurgitation, pulmonary aspiration, and postoperative nausea and vomiting (PONV); no outcome was judged to provide high- or moderate-certainty evidence. Taken together, these findings support a scenario-dependent interpretation of GT placement rather than a universal routine-use or routine-avoidance strategy.

The clinical implication of these findings is that the question “should a gastric tube be placed through the drainage channel?” is not well posed as a single routine-use decision, and is better framed as a scenario-dependent interpretation anchored in the available evidence (Table 3). Three considerations support this reading, and together they account for the structure of the interpretive framework. First, the Tier 1 evidence on gastric insufflation was generated under a specific experimental condition, namely an incremental airway pressure trial with ultrasonographic antral imaging, and should not be mechanically extrapolated to every clinical setting; detection methods across Tier 2 studies varied across ultrasound, epigastric auscultation, and surgeon-rated gastric distension, and these approaches are not interchangeable (53). Second, the Tier 1 evidence on OLP supports the interpretation that GT placement does not materially compromise airway seal, while Tier 2 head-to-head comparisons suggested a device-specific seal–ease trade-off, most apparent with the LMA Protector (45, 46). Some devices were more often associated with easier or faster GT insertion, but the underlying studies were not direct GT-strategy trials and did not support a formal device ranking. Third, for regurgitation, pulmonary aspiration, and PONV, no clear signal was identified across the contributing evidence; however, the rarity of events, the heterogeneity of outcome definitions, and the predominance of indirect evidence mean that absence of a clear signal cannot be equated with proof of absence (54).

These three considerations map onto the interpretive framework as follows: Category (i) is mainly informed by situations in which gastric drainage is clinically meaningful and the available indirect evidence is at least directionally supportive; Category (ii) is mainly informed by low-risk elective contexts in which the absence of a clear advantage signal is reinforced by reported practice variation; and Category (iii) reflects either context-limited evidence or complete evidence gaps. A further consideration cuts across all three categories: high GT insertion success or procedural feasibility, as reported in several Cluster A and Cluster C studies, should not be conflated with demonstrated clinical benefit, and performance, safety, and decision-level evidence retain distinct evidentiary status throughout this synthesis. Several Category (iii) scenarios, including emergency obstetric surgery, prone-position surgery, laparotomy, and populations such as morbidly obese adults, paediatric patients, and patients with full stomach, remain insufficiently informed by the current evidence base.

Regurgitation and pulmonary aspiration are rare perioperative events, and the total number of exposed patients across the available studies was insufficient to provide meaningful precision around these outcomes. The absence of reported aspiration events in the contributing evidence should therefore be interpreted only as the absence of an observed signal in small and heterogeneous studies, not as evidence that GT placement prevents or does not increase aspiration risk. The same caveat applies to regurgitation, where event detection was further constrained by inconsistent operational definitions across studies, and to PONV, which was reported by only two studies in the entire evidence base. These outcomes were rated as Very low certainty under GRADE, and any interpretation of safety in this domain therefore rests on substantially less evidence than is needed to support a safety conclusion. A further methodological consideration is that the detection methods used across the contributing evidence did not measure identical constructs. Ultrasonographic assessment of antral cross-sectional area or derived gastric volume provides a quantitative estimate of gastric distension and is the most objective and reproducible of the available methods (53). Epigastric auscultation detects audible air entry into the stomach during positive-pressure ventilation and is sensitive to the immediate ventilatory event but observer-dependent in interpretation. Surgeon-rated gastric distension reflects intraoperative visual assessment within the surgical field and is influenced by surgical context and observer threshold. Direct observation of air escape through the drainage channel reflects a device-level pathway rather than gastric distension itself. These methods therefore capture overlapping but non-equivalent constructs, with differing sensitivity, specificity, and clinical meaning, and were not interchangeable for synthesis. This measurement heterogeneity contributed to the prespecified decision to synthesize gastric insufflation findings narratively rather than by pooled estimation, and to stratify findings by detection method where data permitted.

This review has several methodological strengths. The evidence base was organized *a priori* into a tiered synthesis that separated direct comparative evidence (Tier 1) from indirect clinical evidence (Tier 2) and from mechanistic and practice-based evidence (Tier 3), which allowed the review to preserve evidentiary boundaries that are often blurred in narrative summaries of mixed study designs. Evidence mapping made the distribution of outcome coverage and evidence gaps explicit, and GRADE was applied only to the clinical evidence tiers for five prespecified outcomes, with downgrade reasoning traceable to the study-level appraisal. The scenario-based interpretive framework was derived from the integrated synthesis and calibrated to evidence certainty. These analytic choices were made to match the observed evidence structure, in which direct comparative data were sparse and outcome coverage was uneven: direct comparative evidence was too sparse for meaningful pooling, outcome definitions and measurement methods were heterogeneous, and a tiered narrative synthesis, evidence map, and interpretive framework were therefore the most appropriate analytic outputs for this review question rather than a retrofit around unavailable quantitative synthesis.

Several limitations temper the conclusions. First, Tier 1 direct evidence was limited to two studies, each informing a single outcome and neither reaching overall low risk of bias, which precluded meta-analysis and any judgement of reproducibility. Second, no included study reached an overall low risk of bias under any applicable tool, and one non-randomized comparative study contributing to Cluster C (22) was assessed at serious risk of bias under ROBINS-I. Third, measurement heterogeneity was substantial for the primary outcome. Gastric insufflation was detected by ultrasonographic antral imaging in some studies and by epigastric auscultation or surgeon-rated distension in others, and these methods differ meaningfully in sensitivity and specificity (53). Fourth, several clinically important rare-event outcomes (regurgitation, pulmonary aspiration, PONV) remained informed by small numbers of events, limiting precise effect estimation (54); the contributing sample sizes were insufficient to exclude clinically meaningful increases in adverse events, and the absence of reported aspiration in the contributing evidence does not constitute evidence of safety in this domain. Fifth, several Category (iii) scenario classifications rested predominantly on indirect or context-limited evidence, as noted above. Finally, twelve reports eligible for full-text retrieval could not be obtained despite repeated attempts; although the quantitative impact of the unretrieved reports is uncertain, the possibility of selective loss of eligible evidence cannot be excluded.

The practical implication of this review is that GT placement through the drainage channel of a second-generation supraglottic airway device is appropriately treated as a scenario-specific clinical decision rather than as a uniform routine-use or avoidance strategy. Such decisions should be informed by the direction and certainty of the available evidence, local institutional policy, and individual clinical judgement. The scenario-based interpretive framework presented in Table 3 is intended to support such interpretation — not to replace formal airway-management guidelines or institutional protocols.

Future research should prioritize trials designed to answer the GT-strategy question directly rather than through device comparison, with three priorities relating to study design, outcome measurement, and scenario targeting. The first priority is direct GT-strategy trials. Specifically, randomized trials are needed in which adults undergoing general anaesthesia with a clearly standardized second-generation SGA are allocated to GT insertion through the drainage channel versus no GT insertion (channel left open or capped). The SGA device, cuff management, and positive-pressure ventilation parameters would need to be held constant between arms, so that the treatment contrast isolates GT strategy rather than device characteristics. A pragmatic primary outcome is ultrasonographic gastric insufflation assessed by change in antral cross-sectional area between a baseline measurement and a post-ventilation measurement, with a prespecified clinical threshold for meaningful change. Key secondary outcomes should comprise patient-level event counts of regurgitation and pulmonary aspiration, postoperative nausea and vomiting captured within 24 hours using a standardized instrument, oropharyngeal leak pressure measured under a standardized manometric protocol, and pharyngolaryngeal morbidity at fixed postoperative time points.

Standardization of measurement is the second priority: routine use of ultrasonographic antral assessment would reduce the detection-method heterogeneity observed in the current evidence base, and consensus operational definitions for regurgitation, pulmonary aspiration, and postoperative nausea and vomiting would support more informative safety inference than *post hoc* reporting (53).

The third priority is scenario targeting, aligned with the Category (iii) evidence gaps identified in this review: emergency obstetric surgery, prone-position surgery, and laparotomy should be addressed first because each is common, clinically important, and currently informed only by context-limited or seriously biased evidence, whereas trials in morbidly obese adults and in patients with full stomach will require careful risk–benefit design but should follow as a second wave (8). Future trials should also be designed to integrate GT-strategy questions within established surgical frameworks such as enhanced recovery after surgery pathways, in which second-generation SGAs are increasingly used and perioperative care components require coordinated evaluation (55).

## 5 Conclusion

In this systematic review with evidence mapping and an interpretive scenario-based framework, direct comparative evidence on gastric tube placement through the drainage channel of second-generation supraglottic airway devices was limited, whereas indirect clinical evidence was broader but heterogeneous in outcome definition and measurement method. GRADE certainty was Low for gastric insufflation and oropharyngeal leak pressure and Very low for regurgitation, pulmonary aspiration, and postoperative nausea and vomiting, with no outcome reaching high or moderate certainty. The pattern most consistent with the available evidence is that gastric tube placement through the drainage channel is appropriately treated as a scenario-specific clinical decision, informed by the direction and certainty of the underlying evidence and the clinical scenario, rather than as a uniform routine-use or avoidance strategy. Future randomized trials comparing gastric tube placement with no gastric tube placement, conducted under clearly standardized second-generation SGA conditions and outcome definitions, are needed to strengthen the evidence base in scenarios and populations currently underrepresented. Until such evidence becomes available, a scenario-based interpretation — neither routine universal placement nor universal avoidance — remains the most evidence-consistent interpretation.

## Data Availability

The original contributions presented in the study are included in the article/Supplementary Material, further inquiries can be directed to the corresponding author.
