# Supplementary material for: Gastric tube placement through the drainage channel of second-generation supraglottic airway devices: a systematic review with evidence mapping
Source: Front Surg. 2026 Jun 9;13:1867470. doi: 10.3389/fsurg.2026.1867470 (PMC13286887; doi:10.3389/fsurg.2026.1867470)
Supplement: Supplementary file 1 [file Datasheet1.pdf]

Supplementary Appendix 1. Full search strategies for bibliographic databases

Review title: Gastric tube placement through the drainage channel of second-generation supraglottic airway devices in adults undergoing general anesthesia: a systematic review with evidence mapping and clinical decision framework

PROSPERO registration: CRD420261368854

Date of search: April 18, 2026

Databases searched: MEDLINE (via PubMed), Embase (via Ovid), Cochrane Central Register of Controlled Trials (CENTRAL), Web of Science Core Collection, Scopus

Language and date restrictions: None applied at the search stage. Databases were searched from inception to April 18, 2026.

Search structure: All database searches used a two-concept Boolean structure combining terms for second-generation supraglottic airway devices (Concept 1) with terms for gastric tube placement or drainage-channel use (Concept 2), using the AND operator. Device and tube terms were searched as free-text in title, abstract, and keyword fields (syntax adapted to each database).

1. MEDLINE (via PubMed)

Search date: April 18, 2026 **Fields searched:** Title/Abstract [tiab]

```
(
   "LMA ProSeal"[tiab]
   OR ProSeal[tiab]
   OR "LMA Supreme"[tiab]
   OR Supreme[tiab]
   OR "i-gel"[tiab]
   OR AuraGain[tiab]
   OR "LMA Protector"[tiab]
   OR "Baska Mask"[tiab]
   OR "LMA Gastro"[tiab]
   OR SaCoVLM[tiab]
)
AND
(
   "gastric tube"[tiab]
   OR "orogastric tube"[tiab]
   OR "nasogastric tube"[tiab]
   OR "gastric drain*"[tiab]
   OR "drainage channel"[tiab]
```

OR "gastric channel"[tiab]
OR "gastric decompression"[tiab]
)

Records retrieved: 211

2. Embase

Search date: April 18, 2026 **Fields searched:** Title, Abstract, Keywords [ti,ab,kw]

('lma proseal':ti,ab,kw OR proseal:ti,ab,kw OR 'lma supreme':ti,ab,kw OR supreme:ti,ab,kw OR 'i-gel':ti,ab,kw OR auragain:ti,ab,kw OR 'lma protector':ti,ab,kw OR 'baska mask':ti,ab,kw OR 'lma gastro':ti,ab,kw OR sacovlm:ti,ab,kw)
AND
('gastric tube':ti,ab,kw OR 'orogastric tube':ti,ab,kw OR 'nasogastric tube':ti,ab,kw OR 'gastric drainage':ti,ab,kw OR 'drainage channel':ti,ab,kw OR 'gastric channel':ti,ab,kw OR 'gastric decompression':ti,ab,kw)

Records retrieved: 361

3. Cochrane Central Register of Controlled Trials (CENTRAL)

Search date: April 18, 2026 **Fields searched:** Default (Title/Abstract/Keyword)

(
"LMA ProSeal"
OR ProSeal
OR "LMA Supreme"
OR Supreme
OR "i-gel"
OR igel
OR AuraGain
OR "LMA Protector"
OR "Baska Mask"
OR "LMA Gastro"
OR SaCoVLM
)
AND
(
"gastric tube"
OR "orogastric tube"
OR "nasogastric tube"
OR "gastric drainage"
OR "drainage channel"
OR "gastric channel"

```
   OR "gastric decompression"
)
```

Records retrieved: 352

4. Web of Science Core Collection

Search date: April 18, 2026 **Field tag:** Topic (TS) — searches title, abstract, author keywords, and Keywords Plus

```
TS=(
   (
      "LMA ProSeal"
      OR ProSeal
      OR "LMA Supreme"
      OR Supreme
      OR "i-gel"
      OR igel
      OR AuraGain
      OR "LMA Protector"
      OR "Baska Mask"
      OR "LMA Gastro"
      OR SaCoVLM
   )
   AND
   (
      "gastric tube"
      OR "orogastric tube"
      OR "nasogastric tube"
      OR "gastric drainage"
      OR "drainage channel"
      OR "gastric channel"
      OR "gastric decompression"
   )
)
```

Records retrieved: 175

5. Scopus

Search date: April 18, 2026 **Field tag:** TITLE-ABS-KEY — searches article title, abstract, and keywords

```
TITLE-ABS-KEY(
   (
```

```
    "LMA ProSeal"
    OR ProSeal
    OR "LMA Supreme"
    OR Supreme
    OR "i-gel"
    OR igel
    OR AuraGain
    OR "LMA Protector"
    OR "Baska Mask"
    OR "LMA Gastro"
    OR SaCoVLM
  )
  AND
  (
    "gastric tube"
    OR "orogastric tube"
    OR "nasogastric tube"
    OR "gastric drainage"
    OR "drainage channel"
    OR "gastric channel"
    OR "gastric decompression"
  )
)
```

Records retrieved:259

Supplementary searches

In addition to database searches, backward citation tracking of all included studies and relevant systematic reviews was performed, and forward citation tracking was performed using Web of Science. When reports lacked sufficient detail to determine GT management strategy or extract outcome data, corresponding authors were contacted by email up to two times over a 4-week period.

Note on search strategy: The search was intentionally structured with a two-concept Boolean combining device names (Concept 1) with gastric-tube and drainage-channel terms (Concept 2), using the AND operator and without additional outcome-based restrictors. This structure was chosen so that studies could be identified regardless of their anticipated tier assignment, with tier allocation performed during full-text screening (see Methods Section 2.5).

Supplementary Table 1. Characteristics of Tier 1 studies providing direct comparative evidence on gastric tube placement through the drainage channel of second-generation supraglottic airway devices (n = 2).

Study ID	Reference	First author	Year	Country	Study design	Population	Sample size (n)	Surgical setting	Airway device (s)	Gastric tube / drainage strategy	Relevant outcomes reported	Key findings relevant to this review	Evidence tier
**Freisburger_2006**	Freisburger et al. Der Anaesthesist. 2006;55:1255-1258.	Freisburger	2006	Germany	Before–after clinical study	Adults, ASA I-III	98	Elective eye surgery under total intravenous anesthesia	LMA ProSeal	Airway leak pressure measured before and after gastric tube placement through the drain tube	Airway leak pressure	Airway leak pressure did not change significantly after gastric tube placement through the ProSeal drain tube (25 vs 25 cmH2O, p = 0.6), supporting that GT placement does not materially compromise airway seal.	Tier 1
**Hell_2024**	Hell et al.	Hell	2024	Germ	Randomi	Adults,	152	General	LMA	Within-patient	Gastric	Inserting a	Tier 1

Study ID	Reference	First author	Year	Country	Study design	Population	Sample size (n)	Surgical setting	Airway device (s)	Gastric tube / drainage strategy	Relevant outcomes reported	Key findings relevant to this review	Evidence tier
	Journal of Clinical Anesthesia. 2024;99:111653.			any	zed crossover trial	ASA I-III		anesthesia in a tertiary academic hospital	ProSeal (inflatable cuff) vs i-gel (thermoplastic elastomer cuff)	comparison of ventilation with vs without an inserted gastric tube during pressure and leak tests	insufflation; OLP testing; gastric channel flow; PONV	gastric tube increased gastric insufflation (10.9% with GT vs 2.7% without GT; p = 0.009) during positive-pressure ventilation. Cuff design (inflatable vs thermoplastic elastomer) did not alter this effect.	

Studies are presented in chronological order by year of publication. Tier 1 comprised studies directly comparing gastric tube insertion versus non-insertion through the drainage channel of a second-generation supraglottic airway device. Key quantitative findings are reported where available. Full risk-of-bias assessments for these studies are provided in Supplementary Table 4.

Abbreviations: ASA, American Society of Anesthesiologists; GT, gastric tube; LMA, laryngeal mask airway; OLP, oropharyngeal leak pressure; PONV, postoperative nausea and vomiting; SGA, supraglottic airway.

Supplementary Figure 1. Risk-of-bias assessment of clinical studies included in the systematic review of gastric tube placement through second-generation supraglottic airway devices.

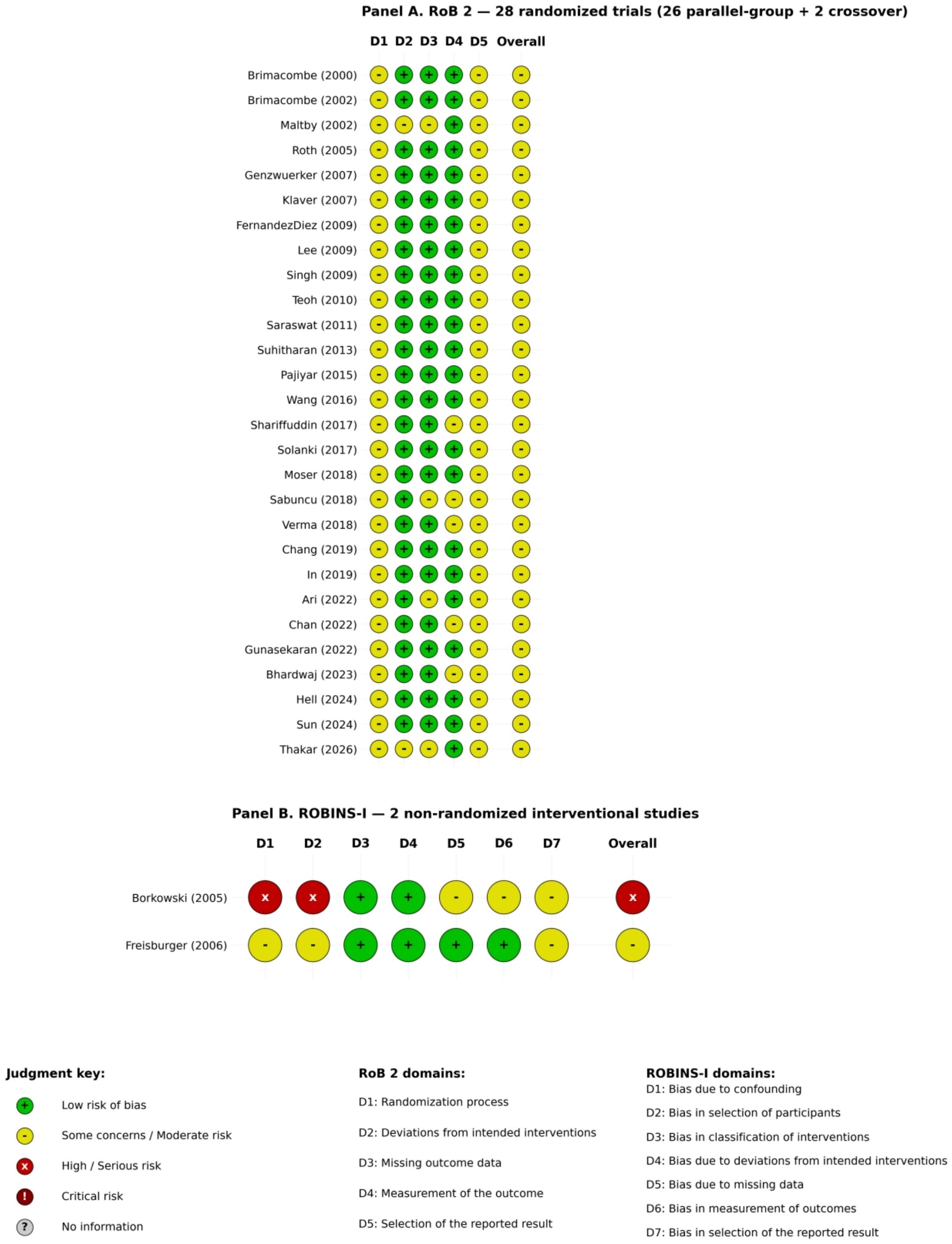

Panel A shows the Cochrane RoB 2 assessment of 28 randomized trials, comprising 26 parallel-group randomized controlled trials and 2 crossover randomized controlled trials. For the two crossover trials (Brimacombe 2000 and Hell 2024), the RoB 2 extension for crossover

designs was applied; the D1 column additionally reflects the period and carryover considerations specific to crossover designs.

Panel B shows the ROBINS-I assessment of 2 non-randomized interventional studies, comprising 1 before–after study (Freisburger 2006) and 1 non-randomized comparative study (Borkowski 2005).

Symbols within each circle represent the domain-level judgment: + (green), low risk of bias; − (yellow), some concerns (RoB 2) or moderate risk (ROBINS-I); × (red), high risk of bias (RoB 2) or serious risk (ROBINS-I); **!** (dark red), critical risk (ROBINS-I only); **?** (grey), no information. The "Overall" column reflects the consolidated reviewer consensus for each study. All assessments were conducted independently by two reviewers (XRY and SJD) with complete agreement (100% concordance) achieved before consensus discussion. Detailed domain-level justifications for every included study are provided in Supplementary Appendix 3.

Abbreviations: RoB 2, Cochrane Risk of Bias 2 tool; ROBINS-I, Risk Of Bias In Non-randomized Studies – of Interventions.

Supplementary Table 2. Consolidated risk-of-bias and methodological appraisal summary for all 36 studies included in the systematic review of gastric tube placement through second-generation supraglottic airway devices.

Study	Evidence tier	Tier 2 cluster	Assessment tool	Overall judgment	Main reason	Brief justification
**Brimacombe (2002)**	Tier 2	A	RoB 2	Some concerns	allocation concealment unclear	The article states that airway-device and orogastric-tube randomization was done by opening a sealed envelope, but opacity/custody of envelopes was not reported; intraoperative data were collected by unblinded observers.
**Maltby (2002)**	Tier 2	B	RoB 2	Some concerns	analysis population unclear	Four obese patients crossed from the ProSeal group to endotracheal intubation and were excluded from the analysis, so the analyzed population departs from the randomized allocation and is not clearly intention-to-treat.
**Roth (2005)**	Tier 2	B	RoB 2	Some concerns	allocation concealment unclear	Patients were randomized by opening a sealed envelope, but the report does not describe sequence generation or who controlled concealment before allocation.
**Genzwuerker (2007)**	Tier 2	A	RoB 2	Some concerns	allocation concealment unclear	Allocation was by sealed envelope immediately before induction, yet the paper does not clarify how the sequence was

Study	Evidence tier	Tier 2 cluster	Assessment tool	Overall judgment	Main reason	Brief justification
						generated or who safeguarded envelopes before opening.
**Klaver (2007)**	Tier 2	A	RoB 2	Some concerns	analysis population unclear	The study protocol was abandoned after failed insertion or ventilation and alternative airway management was used, but it is not clear how those post-randomization failures were handled in the comparative analysis.
**FernandezDiez (2009)**	Tier 2	A	RoB 2	Some concerns	randomization process insufficiently described	The study is described as randomized, but the current full-text extract available in the project does not provide article-specific details on sequence generation or allocation concealment.
**Lee (2009)**	Tier 2	A	RoB 2	Some concerns	allocation concealment unclear	Patients were randomized with sealed opaque envelopes and postoperative symptoms were assessed by a blinded observer, but the process that generated and protected the allocation sequence before induction is not fully described.
**Singh (2009)**	Tier 2	A	RoB 2	Some concerns	randomization process insufficiently described	The report states a randomized comparison, but current project files do not capture a clear description of sequence generation or concealment methods.

Study	Evidence tier	Tier 2 cluster	Assessment tool	Overall judgment	Main reason	Brief justification
**Teoh (2010)**	Tier 2	A	RoB 2	Some concerns	no accessible prespecified analysis plan / trial registration	Computer-generated random numbers, sealed opaque envelopes, participant blinding, and a blinded postoperative observer were described, but no accessible protocol or analysis plan was available to verify the prespecified outcome hierarchy.
**Saraswat (2011)**	Tier 2	B	RoB 2	Some concerns	allocation concealment unclear	Computer-generated random assignment in opaque envelopes was reported, but the paper does not fully detail who generated the sequence and how envelope concealment was maintained until allocation.
**Suhitharan (2013)**	Tier 2	C	RoB 2	Some concerns	no accessible prespecified analysis plan / trial registration	Computer-generated randomization, sealed opaque envelopes, participant blinding, and blinded postoperative assessment were described, but no accessible protocol or registry-based analysis plan was available to verify prespecified reporting.
**Pajiyar (2015)**	Tier 2	A	RoB 2	Some concerns	selective emphasis on secondary outcomes	The trial was randomized and registered, but GT-related outcomes were secondary to airway seal pressure and the current files do not include a prespecified analysis plan clarifying how these secondary outcomes were prioritized.

Study	Evidence tier	Tier 2 cluster	Assessment tool	Overall judgment	Main reason	Brief justification
**Wang (2016)**	Tier 2	A	RoB 2	Some concerns	randomization process insufficiently described	The study is treated as a randomized parallel comparison in the project, but the extracted full-text material does not clearly report sequence generation, allocation concealment, or prespecified analysis details.
**Shariffuddin (2017)**	Tier 2	A	RoB 2	Some concerns	no accessible prespecified analysis plan / trial registration	Randomization, sealed opaque allocation, patient blinding, blinded postoperative assessment, and prospective registration were described, but no accessible statistical analysis plan was available to confirm prespecified outcome selection.
**Solanki (2017)**	Tier 2	A	RoB 2	Some concerns	no accessible prespecified analysis plan / trial registration	Computer-generated randomization, opaque sealed allocation, blinded fibreoptic assessment, and CTRI registration were reported, but the current project files do not contain a prespecified analysis plan.
**Moser (2018)**	Tier 2	A	RoB 2	Some concerns	selective emphasis on secondary outcomes	This randomized, registered trial was primarily powered for OLP at a fixed cuff volume; gastric-tube and drainage-related findings relevant to this review were secondary outcomes, and the current project files do not include a protocol specifying their analysis priority.

Study	Evidence tier	Tier 2 cluster	Assessment tool	Overall judgment	Main reason	Brief justification
**Sabuncu (2018)**	Tier 2	C	RoB 2	Some concerns	analysis population unclear	The trial was registered and envelope-allocated, but the current project materials are internally inconsistent about the recruited and analyzed numbers across the three arms, making the analysis population difficult to reconstruct.
**Verma (2018)**	Tier 2	A	RoB 2	Some concerns	outcome measurement / assessor blinding insufficiently described	The trial included subjective or assessor-sensitive endpoints such as sore throat and hoarseness in addition to OGT passage, but blinded outcome assessment is not clearly described in the current files.
**Chang (2019)**	Tier 2	A	RoB 2	Some concerns	outcome measurement / assessor blinding insufficiently described	The investigators who inserted the device, assessed airway leak pressure, and graded gastric-tube placement were explicitly unblinded, so assessor-sensitive secondary outcomes cannot be assumed protected from measurement bias.
**In (2019)**	Tier 2	B	RoB 2	Some concerns	allocation concealment unclear	Online randomization software and a non-translucent envelope were used, but the researcher who inserted the device opened the envelope just before induction, leaving allocation concealment procedures not fully independent from intervention delivery.

Study	Evidence tier	Tier 2 cluster	Assessment tool	Overall judgment	Main reason	Brief justification
**Ari (2022)**	Tier 2	A	RoB 2	Some concerns	missing outcome data handling insufficiently described	The analyzed sample was 62 with small post-allocation losses or exclusions in the current project files, but the handling of these missing data and the final analysis set is not fully described.
**Chan (2022)**	Tier 2	A	RoB 2	Some concerns	outcome measurement / assessor blinding insufficiently described	Post-insertion performance and postoperative sore throat were assessed in a single-blinded trial, but the current full text does not clearly show that assessors for all review-relevant outcomes were blinded.
**Gunasekaran (2022)**	Tier 2	B	RoB 2	Some concerns	no accessible prespecified analysis plan / trial registration	Block randomization, SNOSE allocation concealment, and CTRI registration were reported, but no accessible prespecified statistical analysis plan was available in the project files.
**Bhardwaj (2023)**	Tier 2	A	RoB 2	Some concerns	outcome measurement / assessor blinding insufficiently described	Random sequence generation, opaque-envelope allocation, registry entry, and no dropout were reported, but several review-relevant outcomes depended on operator or postoperative assessment and blinded outcome assessment is not clearly documented.
**Sun (2024)**	Tier 2	C	RoB 2	Some	no accessible prespecified	Computer-generated allocation, sealed

Study	Evidence tier	Tier 2 cluster	Assessment tool	Overall judgment	Main reason	Brief justification
				concerns	analysis plan / trial registration	opaque envelopes, prospective registration, and CONSORT-style reporting were described, but no detailed prespecified statistical analysis plan was accessible in the current project files.
**Thakar (2026)**	Tier 2	A	RoB 2	Some concerns	randomization process insufficiently described	The article describes itself as a 'prospective randomized observational study', and the method section does not clearly resolve sequence generation, concealment, or the exact randomized analysis set.
**Brimacombe (2000)**	Tier 2	A	RoB 2 crossover extension	Some concerns	period effects and analysis appropriateness unclear in a crossover design	Both devices were assessed within the same anesthetic episode, but order/period handling and crossover analysis details are incompletely reported; the introducer-related substudy also complicates interpretation.
**Hell (2024)**	Tier 1	NaN	RoB 2 crossover extension	Some concerns	period/carryover effects and prespecified analysis handling insufficiently described	This randomized crossover study addressed a directly relevant GT question with mainly objective outcomes, but the current project files do not fully show how sequence, period effects, carryover, and prespecified crossover analyses were handled.
**Borkowski (2005)**	Tier 2	C	ROBINS-I	Serious risk	non-randomized airway allocation with likely	Allocation to PLMA versus tracheal tube appears clinician-selected rather than

Study	Evidence tier	Tier 2 cluster	Assessment tool	Overall judgment	Main reason	Brief justification
					confounding and participant-selection bias	randomized in abdominal surgery, and recruitment-flow plus missing-data handling are incompletely reported, leaving substantial residual confounding.
**Freisburger (2006)**	Tier 1	NaN	ROBINS-I	Moderate risk	before-after within-patient comparison remains vulnerable to order/time confounding	Leak pressure before versus after gastric tube placement was measured in the same patients, with clearly defined interventions and objective outcomes, but confounding by sequence, time, and unreported prespecified analysis remains plausible.
**Evans (2002)**	Tier 2	A	JBI case series	Moderate limitations	consecutive and complete case inclusion not clearly reported in an early clinical series	The series provides useful ProSeal and GT-related clinical data with standard outcome reporting, but explicit inclusion criteria, recruitment flow, and confirmation of complete case capture are not fully available in the current files.
**Evans (2005)**	Tier 2	C	JBI case series	Moderate limitations	single-arm postpartum series with unclear recruitment completeness	GT placement, gastric aspirate, and airway outcomes are clearly reported, but the extract does not show whether all eligible postpartum tubal-ligation cases were enrolled consecutively or whether any cases were omitted.
**Brimacombe**	Tier 2	C	JBI case series	Moderate	retrospective audit with unclear	The prone-position ProSeal audit reports

Study	Evidence tier	Tier 2 cluster	Assessment tool	Overall judgment	Main reason	Brief justification
**(2007)**				limitations	case ascertainment and completeness of inclusion	clinically relevant GT and gastric-safety information, but formal inclusion criteria and full audit capture are not clearly described in the current project files.
**Li (2017)**	Tier 2	C	JBI case series	Moderate limitations	large single-arm cohort with unclear consecutive enrollment	The SLMA plus orogastric tube strategy and aspiration surveillance are well described, but the current files do not confirm that all eligible category 2/3 cesarean cases were included consecutively without selection.
**Liu (2021)**	Tier 2	B	JBI case series	Moderate limitations	multicenter single-arm series with incomplete recruitment-flow reporting	Adults undergoing laparoscopic surgery were clearly defined and outcomes were recorded in a standardized way, but the current project files do not fully document consecutive inclusion, center-level case capture, or missing-data handling.
**Adachi (2024)**	Tier 3	NaN	Structured checklist	Moderate limitations	mechanistic relevance is good, but clinical generalizability and survey representativeness are limited	The manikin procedures and GT outcomes are clearly standardized and relevant to the review question, but confidence is limited by the artificial model and a narrow single-department survey component requiring AXIS-style caution.

Studies are grouped by the assessment tool applied and, within each tool, presented in chronological order by publication year. The Evidence tier column reflects the prespecified three-tier evidence framework (Methods 2.3.1). The Tier 2 cluster column identifies thematic sub-grouping within Tier 2, in which Cluster A denotes studies primarily investigating gastric tube and drainage-channel performance, Cluster B denotes studies reporting gastric-related safety outcomes (gastric insufflation, regurgitation, pulmonary aspiration, or postoperative nausea and vomiting), and Cluster C denotes studies examining special clinical scenarios or context-specific device management issues. The Overall judgment column reflects the consolidated reviewer consensus for each study. All assessments were conducted independently by two reviewers (XRY and SJD) with complete agreement (100% concordance) achieved before consensus discussion. Domain-level judgments for RoB 2 and ROBINS-I assessments are visualized in Supplementary Figure 1 and are justified in detail in Supplementary Appendix 3; JBI case-series domain-level assessments are provided in Supplementary Appendix 2A; and Tier 3 structured-checklist and AXIS-informed assessment items are provided in Supplementary Appendix 2B.

Abbreviations: AXIS, Appraisal tool for Cross-Sectional Studies; JBI, Joanna Briggs Institute; RoB 2, Cochrane Risk of Bias 2 tool; ROBINS-I, Risk Of Bias In Non-randomized Studies – of Interventions.

Supplementary Appendix 2A. Joanna Briggs Institute (JBI) critical appraisal checklist for case series: item-level methodological appraisal of the 5 single-arm clinical studies included in the systematic review.

Study	Q1 — Clear inclusion criteria	Q2 — Condition measured reliably	Q3 — Valid identification methods	Q4 — Consecutive inclusion	Q5 — Complete inclusion	Q6 — Demographics reported	Q7 — Clinical information reported	Q8 — Outcomes/follow-up reported	Q9 — Site demographics reported	Q10 — Statistical analysis appropriate	Overall judgment
**Evans (2002)**	Unclear	Yes	Yes	Unclear	Unclear	Yes	Yes	Yes	Yes	Yes	Moderate limitations
**Evans (2005)**	Yes	Yes	Yes	Unclear	Unclear	Yes	Yes	Yes	Yes	Yes	Moderate limitations
**Brimacombe (2007)**	Unclear	Yes	Yes	Unclear	Unclear	Yes	Yes	Yes	Yes	Yes	Moderate limitations
**Li (2017)**	Yes	Yes	Yes	Unclear	Unclear	Yes	Yes	Yes	Yes	Yes	Moderate limitations
**Liu (2021)**	Yes	Yes	Yes	Unclear	Unclear	Yes	Yes	Yes	Yes	Yes	Moderate limitations

The five case series comprised routine-use clinical reports and one retrospective audit of second-generation supraglottic airway devices in specific clinical contexts (postpartum tubal ligation, obstetric anaesthesia, prone-position surgery, and laparoscopic surgery). Each of the 10 JBI checklist items was judged independently by two reviewers (XRY and SJD), with possible responses of Yes, No, Unclear, or Not applicable, and complete agreement (100% concordance) was achieved before consensus discussion. The Overall judgment was assigned as "moderate limitations" when recurrent Unclear responses were concentrated in inclusion- and recruitment-related items (particularly Q4 on consecutive inclusion and Q5 on complete inclusion), while other checklist items were adequately reported. These appraisals informed the stratified narrative synthesis of single-arm evidence but did not contribute to pooled effect estimation.

Abbreviations: JBI, Joanna Briggs Institute.

Supplementary Appendix 2B. Methodological appraisal of the Tier 3 study (Adachi et al., 2024) using a prespecified structured checklist for the manikin component and an AXIS-informed assessment for the embedded practice survey component.

Study	Component	Item	Judgment	Note
**Adachi (2024)**	Manikin component (structured checklist)	Model fidelity	Unclear	Commercial airway manikin was used, but fidelity to in vivo GT passage was not formally validated in the report.
**Adachi (2024)**	Manikin component (structured checklist)	Procedural standardization across the four SGAs	Yes	All participants inserted the same four size-4 SGAs with standardized tube depth, head position, and operating-room conditions.
**Adachi (2024)**	Manikin component (structured checklist)	Operator characteristics and training	Yes	Participants were 32 anesthesiologists from the department, each with more than two years of clinical experience.
**Adachi (2024)**	Manikin component (structured checklist)	Outcome measurement (GT insertion time/ease)	Yes	GT insertion time and participant-rated ease were explicitly defined and measured consistently across devices.
**Adachi (2024)**	Manikin component (structured checklist)	Reporting completeness	Unclear	Methods are generally clear, but some reporting detail remains limited, including model-related generalizability and survey methodology.
**Adachi (2024)**	Embedded survey (AXIS-informed)	Study aims clearly stated	Yes	The report clearly states that routine use of second-generation SGAs and GT insertion practice were surveyed.
**Adachi (2024)**	Embedded survey (AXIS-informed)	Study design appropriate for stated aims	Yes	A brief departmental questionnaire was a reasonable descriptive design for documenting local practice patterns.

Study	Component	Item	Judgment	Note
**Adachi (2024)**	Embedded survey (AXIS-informed)	Sample size justified	No	The survey sample reflected participating anesthesiologists, but no formal sample-size justification was reported.
**Adachi (2024)**	Embedded survey (AXIS-informed)	Target/reference population clearly defined	Yes	The target group was clearly the participating anesthesiologists in the department.
**Adachi (2024)**	Embedded survey (AXIS-informed)	Sample frame taken from appropriate population base	Yes	The sample frame was the study department from which all surveyed participants were drawn, although it was narrow.
**Adachi (2024)**	Embedded survey (AXIS-informed)	Selection process likely to yield representative sample	No	Single-department sampling limits representativeness beyond the local practice environment.
**Adachi (2024)**	Embedded survey (AXIS-informed)	Response rate reported and non-responders considered	Yes	All 32 recruited participants completed the questionnaire; a separate non-responder analysis was not needed within the enrolled sample.
**Adachi (2024)**	Embedded survey (AXIS-informed)	Outcome measures and classifications appropriate for aims	Yes	The questionnaire addressed routine device choice and frequency of GT insertion with clear categorical reporting.

The included Tier 3 study used a dual-component design combining a manikin-based evaluation of gastric tube insertion performance across four second-generation supraglottic airway devices (i-gel, Ambu AuraGain, LMA ProSeal, and LMA Supreme) with an embedded practice survey of 32 anesthesiologists drawn from a single institutional department. The structured checklist (5 items) addressed model fidelity, procedural standardization across the four devices, operator characteristics, outcome measurement for gastric tube insertion, and reporting completeness. The AXIS-informed assessment (8 items) addressed stated aims, study design, sample size justification, target population definition, sampling frame appropriateness, representativeness of selection, reporting of response rate and non-response, and appropriateness of outcome measures. Each item was judged as Yes, No, or Unclear independently by two reviewers (XRY and SJD), with complete agreement achieved before consensus discussion. Tier 3 evidence contributed to feasibility assessment,

characterization of device-related failure modes, and description of practice variation, and informed the scenario-based clinical decision framework, but was not used for effect estimation alongside Tier 1 and Tier 2 clinical evidence.

Abbreviations: AXIS, Appraisal tool for Cross-Sectional Studies; GT, gastric tube; SGA, supraglottic airway.

Supplementary Appendix 3. Domain-level risk-of-bias assessments for 28 randomized trials (Cochrane RoB 2) and 2 non-randomized interventional studies (ROBINS-I), with consolidated main reasons and brief justifications for each study.

Section	Study	Domain 1	Domain S (crossover only)	Domain 2	Domain 3	Domain 4	Domain 5	Domain 6	Domain 7	Overall	Main reason	Justification
**A. RoB 2 (parallel RCTs)**	Brimacombe (2002)	Some concerns	—	Low	Low	Low	Some concerns	—	—	Some concerns	allocation concealment unclear	The article states that airway-device and orogastric-tube randomization was done by opening a sealed envelope, but opacity/custody of envelopes was not reported; intraoperative data were collected by unblinded observers.
**A. RoB 2 (parallel RCTs)**	Maltby (2002)	Some concerns	—	Some concerns	Some concerns	Low	Some concerns	—	—	Some concerns	analysis population unclear	Four obese patients crossed from the ProSeal group to endotracheal intubation and were excluded from the analysis, so the analyzed population departs from the

Section	Study	Domain 1	Domain S (crossover only)	Domain 2	Domain 3	Domain 4	Domain 5	Domain 6	Domain 7	Overall	Main reason	Justification
												randomized allocation and is not clearly intention-to-treat.
**A. RoB 2 (parallel RCTs)**	Roth (2005)	Some concerns	—	Low	Low	Low	Some concerns	—	—	Some concerns	allocation concealment unclear	Patients were randomized by opening a sealed envelope, but the report does not describe sequence generation or who controlled concealment before allocation.
**A. RoB 2 (parallel RCTs)**	Genzwuerker (2007)	Some concerns	—	Low	Low	Low	Some concerns	—	—	Some concerns	allocation concealment unclear	Allocation was by sealed envelope immediately before induction, yet the paper does not clarify how the sequence was generated or who safeguarded envelopes before

Section	Study	Domain 1	Domain S (crossover only)	Domain 2	Domain 3	Domain 4	Domain 5	Domain 6	Domain 7	Overall	Main reason	Justification
												opening.
**A. RoB 2 (parallel RCTs)**	Klaver (2007)	Some concerns	—	Low	Low	Low	Some concerns	—	—	Some concerns	analysis population unclear	The study protocol was abandoned after failed insertion or ventilation and alternative airway management was used, but it is not clear how those post-randomization failures were handled in the comparative analysis.
**A. RoB 2 (parallel RCTs)**	FernandezDiez (2009)	Some concerns	—	Low	Low	Low	Some concerns	—	—	Some concerns	randomization process insufficiently described	The study is described as randomized, but the current full-text extract available in the project does not provide article-specific details on sequence generation or

Section	Study	Domain 1	Domain S (crossover only)	Domain 2	Domain 3	Domain 4	Domain 5	Domain 6	Domain 7	Overall	Main reason	Justification
												allocation concealment.
**A. RoB 2 (parallel RCTs)**	Lee (2009)	Some concerns	—	Low	Low	Low	Some concerns	—	—	Some concerns	allocation concealment unclear	Patients were randomized with sealed opaque envelopes and postoperative symptoms were assessed by a blinded observer, but the process that generated and protected the allocation sequence before induction is not fully described.
**A. RoB 2 (parallel RCTs)**	Singh (2009)	Some concerns	—	Low	Low	Low	Some concerns	—	—	Some concerns	randomization process insufficiently described	The report states a randomized comparison, but current project files do not capture a clear description of sequence generation or concealment

Section	Study	Domain 1	Domain S (crossover only)	Domain 2	Domain 3	Domain 4	Domain 5	Domain 6	Domain 7	Overall	Main reason	Justification
												methods.
**A. RoB 2 (parallel RCTs)**	Teoh (2010)	Some concerns	—	Low	Low	Low	Some concerns	—	—	Some concerns	no accessible prespecified analysis plan / trial registration	Computer-generated random numbers, sealed opaque envelopes, participant blinding, and a blinded postoperative observer were described, but no accessible protocol or analysis plan was available to verify the prespecified outcome hierarchy.
**A. RoB 2 (parallel RCTs)**	Saraswat (2011)	Some concerns	—	Low	Low	Low	Some concerns	—	—	Some concerns	allocation concealment unclear	Computer-generated random assignment in opaque envelopes was reported, but the paper does not fully detail who generated the sequence and how envelope

Section	Study	Domain 1	Domain S (crossover only)	Domain 2	Domain 3	Domain 4	Domain 5	Domain 6	Domain 7	Overall	Main reason	Justification
												concealment was maintained until allocation.
**A. RoB 2 (parallel RCTs)**	Suhitharan (2013)	Some concerns	—	Low	Low	Low	Some concerns	—	—	Some concerns	no accessible prespecified analysis plan / trial registration	Computer-generated randomization, sealed opaque envelopes, participant blinding, and blinded postoperative assessment were described, but no accessible protocol or registry-based analysis plan was available to verify prespecified reporting.
**A. RoB 2 (parallel RCTs)**	Pajiyar (2015)	Some concerns	—	Low	Low	Low	Some concerns	—	—	Some concerns	selective emphasis on secondary outcomes	The trial was randomized and registered, but GT-related outcomes were secondary to airway

Section	Study	Domain 1	Domain S (crossover only)	Domain 2	Domain 3	Domain 4	Domain 5	Domain 6	Domain 7	Overall	Main reason	Justification
												seal pressure and the current files do not include a prespecified analysis plan clarifying how these secondary outcomes were prioritized.
**A. RoB 2 (parallel RCTs)**	Wang (2016)	Some concerns	—	Low	Low	Low	Some concerns	—	—	Some concerns	randomization process insufficiently described	The study is treated as a randomized parallel comparison in the project, but the extracted full-text material does not clearly report sequence generation, allocation concealment, or prespecified analysis details.
**A. RoB 2 (parallel RCTs)**	Shariffuddin (2017)	Some concerns	—	Low	Low	Some concerns	Some concerns	—	—	Some concerns	no accessible prespecified analysis plan / trial	Randomization, sealed opaque allocation, patient

Section	Study	Domain 1	Domain S (crossover only)	Domain 2	Domain 3	Domain 4	Domain 5	Domain 6	Domain 7	Overall	Main reason	Justification
											registration	blinding, blinded postoperative assessment, and prospective registration were described, but no accessible statistical analysis plan was available to confirm prespecified outcome selection.
A. RoB 2 (parallel RCTs)	Solanki (2017)	Some concerns	—	Low	Low	Low	Some concerns	—	—	Some concerns	no accessible prespecified analysis plan / trial registration	Computer-generated randomization, opaque sealed allocation, blinded fibreoptic assessment, and CTRI registration were reported, but the current project files do not contain a prespecified analysis plan.
A. RoB 2	Moser (2018)	Some	—	Low	Low	Low	Some	—	—	Some	selective emphasis	This randomized,

Section	Study	Domain 1	Domain S (crossover only)	Domain 2	Domain 3	Domain 4	Domain 5	Domain 6	Domain 7	Overall	Main reason	Justification
(parallel RCTs)		concerns					concerns			concerns	on secondary outcomes	registered trial was primarily powered for OLP at a fixed cuff volume; gastric-tube and drainage-related findings relevant to this review were secondary outcomes, and the current project files do not include a protocol specifying their analysis priority.
A. RoB 2 (parallel RCTs)	Sabuncu (2018)	Some concerns	—	Low	Some concerns	Some concerns	Some concerns	—	—	Some concerns	analysis population unclear	The trial was registered and envelope-allocated, but the current project materials are internally inconsistent about the recruited and analyzed numbers across the three

Section	Study	Domain 1	Domain S (crossover only)	Domain 2	Domain 3	Domain 4	Domain 5	Domain 6	Domain 7	Overall	Main reason	Justification
												arms, making the analysis population difficult to reconstruct.
**A. RoB 2 (parallel RCTs)**	Verma (2018)	Some concerns	—	Low	Low	Some concerns	Some concerns	—	—	Some concerns	outcome measurement / assessor blinding insufficiently described	The trial included subjective or assessor-sensitive endpoints such as sore throat and hoarseness in addition to OGT passage, but blinded outcome assessment is not clearly described in the current files.
**A. RoB 2 (parallel RCTs)**	Chang (2019)	Some concerns	—	Low	Low	Low	Some concerns	—	—	Some concerns	outcome measurement / assessor blinding insufficiently described	The investigators who inserted the device, assessed airway leak pressure, and graded gastric-tube placement were explicitly

Section	Study	Domain 1	Domain S (crossover only)	Domain 2	Domain 3	Domain 4	Domain 5	Domain 6	Domain 7	Overall	Main reason	Justification
												unblinded, so assessor-sensitive secondary outcomes cannot be assumed protected from measurement bias.
**A. RoB 2 (parallel RCTs)**	In (2019)	Some concerns	—	Low	Low	Low	Some concerns	—	—	Some concerns	allocation concealment unclear	Online randomization software and a non-translucent envelope were used, but the researcher who inserted the device opened the envelope just before induction, leaving allocation concealment procedures not fully independent from intervention delivery.
**A. RoB 2 (parallel**	Ari (2022)	Some concerns	—	Low	Some concerns	Low	Some concerns	—	—	Some concerns	missing outcome data handling	The analyzed sample was 62 with

Section	Study	Domain 1	Domain S (crossover only)	Domain 2	Domain 3	Domain 4	Domain 5	Domain 6	Domain 7	Overall	Main reason	Justification
RCTs)											insufficiently described	small post-allocation losses or exclusions in the current project files, but the handling of these missing data and the final analysis set is not fully described.
A. RoB 2 (parallel RCTs)	Chan (2022)	Some concerns	—	Low	Low	Some concerns	Some concerns	—	—	Some concerns	outcome measurement / assessor blinding insufficiently described	Post-insertion performance and postoperative sore throat were assessed in a single-blinded trial, but the current full text does not clearly show that assessors for all review-relevant outcomes were blinded.
A. RoB 2 (parallel RCTs)	Gunasekaran (2022)	Some concerns	—	Low	Low	Low	Some concerns	—	—	Some concerns	no accessible prespecified analysis plan / trial	Block randomization, SNOSE allocation

Section	Study	Domain 1	Domain S (crossover only)	Domain 2	Domain 3	Domain 4	Domain 5	Domain 6	Domain 7	Overall	Main reason	Justification
											registration	concealment, and CTRI registration were reported, but no accessible prespecified statistical analysis plan was available in the project files.
**A. RoB 2 (parallel RCTs)**	Bhardwaj (2023)	Some concerns	—	Low	Low	Some concerns	Some concerns	—	—	Some concerns	outcome measurement / assessor blinding insufficiently described	Random sequence generation, opaque-envelope allocation, registry entry, and no dropout were reported, but several review-relevant outcomes depended on operator or postoperative assessment and blinded outcome assessment is not clearly documented.
**A. RoB 2**	Sun (2024)	Some	—	Low	Low	Low	Some	—	—	Some	no accessible	Computer-generated

Section	Study	Domain 1	Domain S (crossover only)	Domain 2	Domain 3	Domain 4	Domain 5	Domain 6	Domain 7	Overall	Main reason	Justification
**(parallel RCTs)**		concerns					concerns			concerns	prespecified analysis plan / trial registration	allocation, sealed opaque envelopes, prospective registration, and CONSORT-style reporting were described, but no detailed prespecified statistical analysis plan was accessible in the current project files.
**A. RoB 2 (parallel RCTs)**	Thakar (2026)	Some concerns	—	Some concerns	Some concerns	Low	Some concerns	—	—	Some concerns	randomization process insufficiently described	The article describes itself as a 'prospective randomized observational study', and the method section does not clearly resolve sequence generation, concealment, or the exact randomized

Section	Study	Domain 1	Domain S (crossover only)	Domain 2	Domain 3	Domain 4	Domain 5	Domain 6	Domain 7	Overall	Main reason	Justification
												analysis set.
**A. RoB 2 (crossover RCTs)**	Brimacombe (2000)	Some concerns	Some concerns	Low	Low	Low	Some concerns	—	—	Some concerns	period effects and analysis appropriateness unclear in a crossover design	Both devices were assessed within the same anesthetic episode, but order/period handling and crossover analysis details are incompletely reported; the introducer-related substudy also complicates interpretation.
**A. RoB 2 (crossover RCTs)**	Hell (2024)	Some concerns	Some concerns	Low	Low	Low	Some concerns	—	—	Some concerns	period/carryover effects and prespecified analysis handling insufficiently described	This randomized crossover study addressed a directly relevant GT question with mainly objective outcomes, but the current project files do not fully show

Section	Study	Domain 1	Domain S (crossover only)	Domain 2	Domain 3	Domain 4	Domain 5	Domain 6	Domain 7	Overall	Main reason	Justification
												how sequence, period effects, carryover, and prespecified crossover analyses were handled.
**B. ROBINS-I**	Borkowski (2005)	Serious	—	Serious	Low	Low	Moderate	Moderate	Moderate	Serious risk	non-randomized airway allocation with likely confounding and participant-selection bias	Allocation to PLMA versus tracheal tube appears clinician-selected rather than randomized in abdominal surgery, and recruitment-flow plus missing-data handling are incompletely reported, leaving substantial residual confounding.
**B. ROBINS-I**	Freisburger (2006)	Moderate	—	Moderate	Low	Low	Low	Low	Moderate	Moderate risk	before-after within-patient	Leak pressure before versus after

Section	Study	Domain 1	Domain S (crossover only)	Domain 2	Domain 3	Domain 4	Domain 5	Domain 6	Domain 7	Overall	Main reason	Justification
											comparison remains vulnerable to order/time confounding	gastric tube placement was measured in the same patients, with clearly defined interventions and objective outcomes, but confounding by sequence, time, and unreported prespecified analysis remains plausible.

Section A presents the RoB 2 domain-level judgments. For the 26 parallel-group randomized controlled trials, the five standard RoB 2 domains were assessed: D1 (Randomization process), D2 (Deviations from intended interventions), D3 (Missing outcome data), D4 (Measurement of the outcome), and D5 (Selection of the reported result). For the 2 crossover randomized trials (Brimacombe 2000 and Hell 2024), the RoB 2 crossover extension was applied, adding Domain S (Period and carryover effects) alongside the five standard domains. Possible domain-level responses were Low, Some concerns, or High.

Section B presents the ROBINS-I domain-level judgments for the 2 non-randomized interventional studies. The seven ROBINS-I domains were assessed: D1 (Bias due to confounding), D2 (Bias in selection of participants), D3 (Bias in classification of interventions), D4 (Bias due to deviations from intended interventions), D5 (Bias due to missing data), D6 (Bias in measurement of outcomes), and D7 (Bias in selection of the reported result). Possible domain-level responses were Low, Moderate, Serious, Critical, or No information.

All assessments were conducted independently by two reviewers (XRY and SJD) with complete agreement (100% concordance) achieved before consensus discussion. A visual summary of the domain-level judgments tabulated here is provided in Supplementary Figure 1. Cells marked "—" indicate a domain not applicable to the respective tool (for example, Domain S is applicable only to the two crossover randomized trials, and Domains D6 and D7 are applicable only to ROBINS-I assessments).

Abbreviations: RoB 2, Cochrane Risk of Bias 2 tool; ROBINS-I, Risk Of Bias In Non-randomized Studies – of Interventions.

Supplementary Table 3. Characteristics of Tier 2 studies providing indirect clinical evidence on gastric tube placement through the drainage channel of second-generation supraglottic airway devices (n = 33)

Study ID	Reference	First author	Year	Country	Study design	Population	Sample size (n)	Surgical setting	Airway device (s)	Gastric tube / drainage strategy	Relevant outcomes reported	Key findings relevant to this review	Evidence tier	Tier 2 thematic subgroup
**Brimacombe_2000**	Brimacombe et al. Anesthesiology. 2000;93:104-109.	Brimacombe	2000	Australia and Austria	Randomized crossover trial	Adults under general anesthesia	120	Minor peripheral surgery under general anesthesia	ProSeal LMA vs standard LMA	Gastric tube inserted through ProSeal drain tube when no drain leak was present	Airway seal; fiberoptic position; gastric tube success/time; insertion difficulty	ProSeal provided a better seal and enabled rapid gastric tube placement, but it was harder to insert unless an introducer was used.	Tier 2	A
**Brimacombe_2002**	Brimacombe et al. Anesthesiology. 2002;96:289-295.	Brimacombe	2002	International multicenter	Randomized controlled trial	Adults, ASA I-II	384	Routine minor surgery under general anesthesia	ProSeal LMA vs Classic LMA	Orogastric tube placement in a randomized subset; through drain tube for ProSeal and behind cuff	Insertion success/time; airway seal; OGT success/time; gastric insufflation; postoperative sore throat	ProSeal provided a better seal and easier orogastric tube placement, whereas the Classic LMA was easier and quicker to insert.	Tier 2	A

Study ID	Reference	First author	Year	Country	Study design	Population	Sample size (n)	Surgical setting	Airway device (s)	Gastric tube / drainage strategy	Relevant outcomes reported	Key findings relevant to this review	Evidence tier	Tier 2 thematic subgroup
										for Classic LMA				
**Evans_2002**	Evans et al. British Journal of Anaesthesia. 2002;88:534-539.	Evans	2002	South Africa	Prospective observational study	Adults under general anesthesia	300	General anesthesia in paralysed or spontaneously breathing patients	ProSeal laryngeal mask airway	Gastric tube placement attempted when no drain leak was present; ease and success recorded	Insertion characteristics; airway seal; gastric tube placement; gastric insufflation; sore throat	ProSeal use and gastric tube placement were usually successful in routine practice, and gastric insufflation was not observed in this series.	Tier 2	A
**Genzwuerker_2007**	Genzwuerker et al. Acta Anaesthesiologica Scandinavica. 2007;51:1373-1377.	Genzwuerker	2007	Germany	Randomized controlled trial	Adults undergoing elective surgery	100	Elective surgery with controlled ventilation	Laryngeal Tube Suction II vs LMA-ProSeal	14-gauge gastric tube passed through the specific channel of both devices	Insertion success/time; oxygenation; leak pressure; gastric tube ease; postoperative complaints	LTS II and ProSeal showed similar airway performance, with high gastric tube insertion success in both groups.	Tier 2	A

Study ID	Reference	First author	Year	Country	Study design	Population	Sample size (n)	Surgical setting	Airway device (s)	Gastric tube / drainage strategy	Relevant outcomes reported	Key findings relevant to this review	Evidence tier	Tier 2 thematic subgroup
**Klaver_2007**	Klaver et al. Anaesthesia. 2007;62:723-727.	Klaver	2007	Netherlands	Randomized controlled trial	Adults under general anesthesia	160	Elective surgery in anesthetized patients	Laryngeal Tube S vs ProSeal LMA	Gastric tube placement through device-specific channel with success/failure recorded	Insertion success/time; leak pressure; gastric tube success; dysphagia; complications	Airway performance was similar, but gastric tube placement succeeded more often with the Laryngeal Tube S than with ProSeal.	Tier 2	A
**Fernandez Diez_2009**	Fernandez Diez et al. Revista Espanola de Anestesiologia y Reanimacion. 2009;56:474-478.	Fernandez Diez	2009	Spain	Randomized controlled trial	Adults undergoing ambulatory surgery	85	General anesthesia with mechanical ventilation and no neuromuscular block	Supreme LMA vs i-gel	Conditions for nasogastric tube passage assessed after device placement	Insertion ease; seal pressure; ventilatory variables; nasogastric tube insertion; side effects	Both devices ventilated similarly, but nasogastric tube passage was easier and faster with the Supreme device than with i-gel.	Tier 2	A
**Lee_2009**	Lee et al.	Lee	2009	Singap	Randomiz	Women,	70	Gynecolog	LMA	14 Fr gastric	OLP; time to	LMA Supreme	Tier 2	A

Study ID	Reference	First author	Year	Country	Study design	Population	Sample size (n)	Surgical setting	Airway device (s)	Gastric tube / drainage strategy	Relevant outcomes reported	Key findings relevant to this review	Evidence tier	Tier 2 thematic subgroup
	Anaesthesia and Intensive Care. 2009;37:815-819.			ore	ed controlled trial	ASA I-II		ical laparoscopic surgery with paralysis	Supreme vs ProSeal LMA	tube inserted via drain tube; insertion time recorded	effective airway; gastric tube insertion time; tidal volume; cuff pressure; complications	allowed faster gastric tube insertion, whereas ProSeal provided a higher leak pressure.		
**Singh_2009**	Singh et al. Indian Journal of Anaesthesia. 2009;53:302-305.	Singh	2009	India	Randomized controlled trial	Adults, ASA I-II	60	Elective surgery under general anesthesia	i-gel vs ProSeal LMA	Ease of gastric tube placement compared between devices	Airway sealing pressure; insertion success; gastric tube ease; airway trauma; regurgitation/ aspiration	i-gel was easier to insert and allowed easier gastric tube placement, whereas ProSeal provided a higher seal pressure.	Tier 2	A
**Teoh_2010**	Teoh et al. Anaesthesia.	Teoh	2010	Singapore	Randomized	Women under	100	Trendelenburg	LMA Supreme	Gastric tube passed	OLP; first-attempt	Leak pressure was similar, but	Tier 2	A

Study ID	Reference	First author	Year	Country	Study design	Population	Sample size (n)	Surgical setting	Airway device (s)	Gastric tube / drainage strategy	Relevant outcomes reported	Key findings relevant to this review	Evidence tier	Tier 2 thematic subgroup
	2010;65:1173-1179.				controlled trial	general anesthesia		gynecological laparoscopy with controlled ventilation	vs i-gel	through each device and insertion time/ease compared	success; GT insertion time/ease; leak fraction; pharyngeal morbidity	gastric tube insertion was easier and faster with LMA Supreme than with i-gel.		
**Pajiyar_2015**	Pajiyar et al. BMC Anesthesiology. 2015;15:69.	Pajiyar	2015	China	Randomized controlled trial	Adults, ASA I-II	80	Elective supine surgery under total intravenous anesthesia	Guardian LMA vs ProSeal LMA	14 Fr gastric tube inserted through gastric port; attempt rate and insertion time recorded	Airway seal; insertion success/time; gastric tube attempt/time; fiberoptic view; postoperative pharyngolaryngeal morbidity	Guardian LMA achieved a higher seal pressure and faster gastric tube insertion than ProSeal, with similar postoperative morbidity.	Tier 2	A
**Wang_2015**	Wang et al. Asian Journal of Surgery.	Wang	2016	China	Randomized controlled trial	Adults, ASA I-II	90	Pelvic surgery under general	LMA Supreme vs i-gel	Gastric tube indwelling time and ease compared	Insertion difficulty; gastric tube indwelling	LMA Supreme allowed easier and faster gastric tube placement,	Tier 2	A

Study ID	Reference	First author	Year	Country	Study design	Population	Sample size (n)	Surgical setting	Airway device (s)	Gastric tube / drainage strategy	Relevant outcomes reported	Key findings relevant to this review	Evidence tier	Tier 2 thematic subgroup
	2016;39:1-5 .							anesthesia		between devices	time/ease; airway seal; PETCO2; peak pressure; complications	whereas i-gel had fewer complications with otherwise similar airway performance.		
**Shariffuddin_2017**	Shariffuddin et al. Anaesthesia and Intensive Care. 2017;45:244-250.	Shariffuddin	2017	Malaysia	Randomized controlled trial	Adults under spontaneous ventilation	100	Elective surgery in a tertiary hospital	Ambu AuraGain vs LMA Supreme Second Seal	14 Fr gastric tube inserted through the gastric drain; ease, success, time, and aspirated volume recorded	OLP; insertion success; insertion time; gastric tube success/ease; sore throat; gastric aspirate volume	AuraGain and LMA Supreme had similar leak pressures, but AuraGain showed better gastric drain functionality and less sore throat.	Tier 2	A
**Solanki_2017**	Solanki et al. Indian Journal of	Solanki	2017	India	Randomized controlled	Overweight or obese	124	Elective surgery in overweigh	ProSeal LMA sized by	Nasogastric tube insertion success	First-attempt success; air leaks; NGT	Selecting ProSeal size by ideal body	Tier 2	A

Study ID	Reference	First author	Year	Country	Study design	Population	Sample size (n)	Surgical setting	Airway device (s)	Gastric tube / drainage strategy	Relevant outcomes reported	Key findings relevant to this review	Evidence tier	Tier 2 thematic subgroup
	Anaesthesia. 2017;61:398-403.				trial	adults, ASA I-II		t and obese patients	actual vs ideal body weight	assessed after size selection strategy	insertion success; fiberoptic view; postoperative complications	weight improved gastric tube insertion and device fit compared with actual body weight.		
**Moser_2018**	Moser et al. Minerva Anestesiologica. 2018;84:684-692.	Moser	2018	Switzerland	Randomized controlled trial	Adult men, ASA I-II	98	Elective surgery in paralyzed adult men	Ambu AuraGain vs LMA Protector	Gastric tube inserted through the designated channel; attempts and gastric aspirate volume documented	OLP; laryngeal mask insertion; gastric tube insertion; intubation time; transdevice intubation resistance	AuraGain was easier to handle overall and had better first-pass gastric tube insertion than LMA Protector.	Tier 2	A
**Verma_2018**	Verma et al. Journal of Anaesthesio	Verma	2018	India	Randomized controlled	Adults, ASA I-II	60	Elective non-laparoscopic	ProSeal LMA vs Larynge	14 Fr orogastric tube passed	Airway seal pressure; peak	ProSeal achieved a better seal and higher orogastric	Tier 2	A

Study ID	Reference	First author	Year	Country	Study design	Population	Sample size (n)	Surgical setting	Airway device (s)	Gastric tube / drainage strategy	Relevant outcomes reported	Key findings relevant to this review	Evidence tier	Tier 2 thematic subgroup
	logy Clinical Pharmacology. 2018;34:58-61.				trial			surgery up to 90 minutes	al Tube Suction	through esophageal suction port and success recorded	pressure; insertion time; OGT passage; sore throat; hoarseness	tube passage success than LTS.		
**Chang_2019**	Chang et al. BMC Anesthesiology. 2019;19:118.	Chang	2019	Korea	Randomized controlled trial	Adults under general anesthesia	110	Elective surgery under general anesthesia	LMA Protector vs i-gel	Gastric tube inserted through gastric channel and graded for ease	Airway leak pressure; insertion time; fiberoptic view; gastric tube ease/failure; blood staining; sore throat	LMA Protector provided a higher seal pressure but made gastric tube placement more difficult than i-gel.	Tier 2	A
**Ari_2022**	Ari et al. Nigerian Journal of Clinical	Ari	2022	Turkey	Randomized controlled trial	Adults, ASA I-III	62	Laparoscopic cholecystectomy	i-gel vs LMA Protector	Gastric tube inserted via drain tube; insertion time	SAD insertion time; GT insertion	i-gel allowed easier and faster gastric tube insertion than	Tier 2	A

Study ID	Reference	First author	Year	Country	Study design	Population	Sample size (n)	Surgical setting	Airway device (s)	Gastric tube / drainage strategy	Relevant outcomes reported	Key findings relevant to this review	Evidence tier	Tier 2 thematic subgroup
	Practice. 2022;25:90-96.									and ease recorded	time/ease; OLP during pneumoperitoneum; postoperative complications	LMA Protector during laparoscopic cholecystectomy.		
**Chan_2022**	Chan et al. Cureus. 2022;14:e23176.	Chan	2022	Malaysia	Randomized controlled trial	Adults aged 18-65 years	60	General anesthesia without neuromuscular blockade	LMA Protector Cuff Pilot vs LMA Supreme	12 Fr gastric tube inserted after placement; insertion attempts recorded	OLP; insertion success/time; laryngeal view; gastric tube first-attempt success; sore throat; blood staining	Overall performance was similar, but first-attempt gastric tube insertion was less successful with LMA Protector than with LMA Supreme.	Tier 2	A
**Bhardwaj_2023**	Bhardwaj et al. Indian Journal of	Bhardwaj	2023	India	Randomized controlled	Adults aged 18-70	90	Anesthetized, paralysed	LMA Protector vs LMA	Gastric tube passed through the	OLP; insertion characteristic	LMA Protector achieved a higher leak	Tier 2	A

Study ID	Reference	First author	Year	Country	Study design	Population	Sample size (n)	Surgical setting	Airway device (s)	Gastric tube / drainage strategy	Relevant outcomes reported	Key findings relevant to this review	Evidence tier	Tier 2 thematic subgroup
	Anaesthesia. 2023;67:S245-S250.				trial	years		patients under elective general anesthesia	ProSeal	gastric access channel or drain tube; failures recorded	s; gastric tube failure; cuff pressure adjustment; blood staining; sore throat	pressure than ProSeal, while gastric tube insertion performance was similar.		
**Thakar_2026**	Thakar et al. International Journal of Medical and Pharmaceutical Research. 2026;7:251-255.	Thakar	2026	India	Prospective comparative study with inconsistent randomization wording; treated as parallel-group RCT for this review.	Adults, ASA I-II	60	Elective surgery under general anesthesia	i-gel vs Ambu AuraGain	Gastric tube insertion attempted after airway placement; ease and number of attempts recorded	Insertion time; OLP; airway sealing quality; gastric tube insertion; hemodynamics; postoperative complications	Both devices were clinically effective, but AuraGain showed better gastric tube insertion performance and a higher seal pressure.	Tier 2	A

Study ID	Reference	First author	Year	Country	Study design	Population	Sample size (n)	Surgical setting	Airway device (s)	Gastric tube / drainage strategy	Relevant outcomes reported	Key findings relevant to this review	Evidence tier	Tier 2 thematic subgroup
**Maltby_2002**	Maltby et al. Canadian Journal of Anesthesia. 2002;49:857-862.	Maltby	2002	Canada	Randomized controlled trial	Adults, ASA I-III, stratified by obesity	109	Laparoscopic cholecystectomy	LMA-ProSeal vs endotracheal tube	14 Fr gastric tube placed in every patient and connected to continuous suction	Pulmonary ventilation; gastric distension; airway pressure; crossover to ETT	In non-obese patients, ProSeal provided ventilation comparable to tracheal intubation without clinically important gastric distension.	Tier 2	B
**Roth_2005**	Roth et al. European Journal of Anaesthesiology. 2005;22:117-122.	Roth	2005	Germany	Randomized controlled trial	Women undergoing gynecological laparoscopy	50	Gynecological laparoscopic surgery with capnoperitoneum	ProSeal LMA vs Laryngeal Tube Suction	Gastric tube inserted if no leak and left in place during surgery	Airway seal; gastric insufflation; gastric tube insertion; airway pressures; regurgitation; postoperative discomfort	Both devices ventilated adequately during gynecological laparoscopy, and no gastric insufflation or regurgitation was detected.	Tier 2	B
**Saraswat_2**	Saraswat et	Sarasw	2011	India	Randomiz	Adults,	60	Laparosco	ProSeal	Nasogastric	NGT	ProSeal with	Tier 2	B

Study ID	Reference	First author	Year	Country	Study design	Population	Sample size (n)	Surgical setting	Airway device (s)	Gastric tube / drainage strategy	Relevant outcomes reported	Key findings relevant to this review	Evidence tier	Tier 2 thematic subgroup
**011**	al. Indian Journal of Anaesthesia. 2011;55:129 -134.	at			ed controlled trial	ASA I-II		pic procedures under general anesthesia	LMA vs endotracheal tube	tube passed in all patients after airway placement	insertion time; oxygenation; ventilation; gastric distention; regurgitation; aspiration; laryngopharyngeal morbidity	routine gastric tube placement provided ventilation comparable to tracheal intubation without gastric distension or aspiration.		
**In_2019**	In et al. Korean Journal of Anesthesiology. 2019;72:39-46.	In	2019	Korea	Randomized controlled trial	Geriatric adults aged 65-85 years	38	Elective surgery expected to last <3 hours	i-gel vs LMA Supreme	Devices with gastric channels; gastric insufflation assessed during leak-pressure testing	Insertion time; ease of insertion; OLP; gastric insufflation; fiberoptic view; ventilator problems	i-gel was easier and faster to insert and was associated with less gastric insufflation than LMA Supreme in geriatric patients.	Tier 2	B
**Liu_2021**	Liu et al.	Liu	2021	China	Prospectiv	Adults	300	Laparosco	LMA	Integrated	Insertion	LMA Protector	Tier 2	B

Study ID	Reference	First author	Year	Country	Study design	Population	Sample size (n)	Surgical setting	Airway device (s)	Gastric tube / drainage strategy	Relevant outcomes reported	Key findings relevant to this review	Evidence tier	Tier 2 thematic subgroup
	BMC Anesthesiology. 2021;21:318.				e observational study	aged 18-70 years		pic surgery	Protector	drainage channel observed for reflux content; device removed and drain inspected after surgery	success; effective ventilation; reflux in drainage channel; aspiration; sore throat; blood staining	showed high insertion and ventilation success in laparoscopy, and reflux seen in the drainage channel was not accompanied by clinical aspiration.		
**Gunasekaran_2022**	Gunasekaran et al. Cureus. 2022;14:e27888.	Gunasekaran	2022	India	Randomized controlled trial	Adults, ASA I-II	120	Elective surgery under general anesthesia	Ambu AuraGain vs ProSeal LMA	Ryle's tube inserted after device placement; gastric antral ultrasound used to estimate gastric volume	Gastric insufflation volume; gastric cross-sectional area; OSP; peak airway pressure; postoperative complications	AuraGain was associated with less gastric insufflation volume than ProSeal and fewer postoperative complications.	Tier 2	B

Study ID	Reference	First author	Year	Country	Study design	Population	Sample size (n)	Surgical setting	Airway device (s)	Gastric tube / drainage strategy	Relevant outcomes reported	Key findings relevant to this review	Evidence tier	Tier 2 thematic subgroup
**Evans_2005**	Evans et al. International Journal of Obstetric Anesthesia. 2005;14:90-95.	Evans	2005	South Africa	Prospective observational study	Women undergoing postpartum tubal ligation	90	Postpartum tubal ligation via minilaparotomy	ProSeal laryngeal mask airway	Gastric tube used to aspirate gastric contents and measure gastric volume/pH	Leak pressure; gastric tube ease; gastric aspirate volume/pH; regurgitation; aspiration; sore throat	ProSeal enabled successful gastric tube placement and gastric aspiration in postpartum tubal ligation, with no suspected regurgitation or aspiration.	Tier 2	C
**Borkowski_2005**	Borkowski et al. Anasthesiol Intensivmed Notfallmed Schmerzther. 2005;40:477-486.	Borkowski	2005	Germany	Non-randomized comparative clinical study	Adults undergoing elective laparotomy	65	Gynecologic or general laparotomy	ProSeal LMA vs tracheal tube	All patients received a gastric tube; insertion time and difficulty were compared	Insertion time/difficulty; gastric tube placement; regurgitation through drain channel; postoperative throat symptoms	ProSeal was feasible for selected laparotomies, and regurgitated fluid could be vented through the drainage channel.	Tier 2	C

Study ID	Reference	First author	Year	Country	Study design	Population	Sample size (n)	Surgical setting	Airway device (s)	Gastric tube / drainage strategy	Relevant outcomes reported	Key findings relevant to this review	Evidence tier	Tier 2 thematic subgroup
**Brimacombe_2007**	Brimacombe et al. Anaesthesia and Intensive Care. 2007;35:222-225.	Brimacombe	2007	Australia and Austria	Retrospective audit	Healthy adults under general anesthesia	245	Prone-position surgery under general anesthesia	ProSeal LMA	Gastric tube inserted after PLMA placement and intermittently suctioned during surgery	Insertion success; gastric tube success; ventilation; regurgitation; gastric insufflation; airway reflex events	In experienced hands, prone ProSeal use was feasible, with successful gastric tube placement and no gastric insufflation or regurgitation.	Tier 2	C
**Suhitharan_2013**	Suhitharan et al. Saudi Journal of Anaesthesia. 2013;7:436-441.	Suhitharan	2013	Singapore	Randomized controlled trial	Women, ASA I-II	70	Ambulatory laparoscopic female sterilization	LMA Supreme vs i-gel	Gastric tube inserted through drain channel, stomach decompressed, insertion time/ease recorded	OLP; insertion success; airway and gastric tube insertion time; leak fraction; pharyngeal morbidity	Both devices provided similar leak pressures, but gastric tube insertion was easier and faster with LMA Supreme than with i-gel.	Tier 2	C
**Li_2017**	Li et al.	Li	2017	China	Prospectiv	Parturie	584	Emergenc	Supreme	Pre-mounted	First-attempt	Supreme LMA	Tier 2	C

Study ID	Reference	First author	Year	Country	Study design	Population	Sample size (n)	Surgical setting	Airway device (s)	Gastric tube / drainage strategy	Relevant outcomes reported	Key findings relevant to this review	Evidence tier	Tier 2 thematic subgroup
	BMC Anesthesiology. 2017;17:169.				e observational study	nts under general anesthesia		y category 2 and 3 cesarean delivery	LMA	14 Fr orogastric tube inserted through the drain tube and suctioned at beginning and end	insertion; OGT insertion success; OLP; regurgitation; aspiration; maternal and neonatal outcomes	and orogastric tube placement were highly successful in this obstetric cohort, with no clinical regurgitation or aspiration.		
**Sabuncu_2018**	Sabuncu et al. Saudi Medical Journal. 2018;39:1082-1089.	Sabuncu	2018	Turkey	Randomized controlled trial	Adults under general anesthesia	105	Elective laparoscopic cholecystectomy	AuraGain vs i-gel vs endotracheal tube	Orogastric tube inserted for drainage; GT/drain capability available in second-generation devices	Ease of insertion; airway pressures; hemodynamics; perioperative complications	AuraGain and i-gel showed similar overall performance in laparoscopic cholecystectomy, but GT-related findings were secondary and limited.	Tier 2	C
**Sun_2024**	Sun et al.	Sun	2024	China	Randomiz	Adults,	70	Lateral	SaCoVL	Gastric tube	OLP;	SaCoVLM	Tier 2	C

Study ID	Reference	First author	Year	Country	Study design	Population	Sample size (n)	Surgical setting	Airway device (s)	Gastric tube / drainage strategy	Relevant outcomes reported	Key findings relevant to this review	Evidence tier	Tier 2 thematic subgroup
	Scientific Reports. 2024;14:2132.				ed controlled trial	ASA I-III		laparoscopic urological surgery	M video LMA vs LMA Supreme	success rate through the drain tube assessed as a secondary outcome	first-attempt success; gastric tube success; alignment; regurgitation/ aspiration; blood staining	provided a higher leak pressure than LMA Supreme, while gastric tube and gastric safety outcomes were secondary measures.		

Studies are grouped by thematic cluster (A, B, or C) and, within each cluster, presented in chronological order by year of publication. Cluster A (n = 20) comprised studies primarily investigating gastric tube and drainage-channel performance. Cluster B (n = 6) comprised studies reporting gastric-related safety outcomes (gastric insufflation, regurgitation, pulmonary aspiration, or postoperative nausea and vomiting). Cluster C (n = 7) comprised studies examining special clinical scenarios, specific patient populations, or context-specific device management issues. Full cluster assignment rationale and risk-of-bias assessments are provided in Supplementary Table 4.

Abbreviations: ASA, American Society of Anesthesiologists; ETT, endotracheal tube; GT, gastric tube; LMA, laryngeal mask airway; LTS, Laryngeal Tube Suction; NGT, nasogastric tube; OGT, orogastric tube; OLP, oropharyngeal leak pressure; OSP, oropharyngeal seal pressure; $PETCO_2$, end-tidal carbon dioxide partial pressure; PLMA, ProSeal laryngeal mask airway; PONV, postoperative nausea and vomiting; SAD, supraglottic airway device; SGA, supraglottic airway; SLMA, Supreme laryngeal mask airway.

Supplementary Table 4. Characteristics of the Tier 3 study providing complementary mechanistic and practice-based evidence on gastric tube placement through the drainage channel of second-generation supraglottic airway devices (n = 1).

Study ID	Reference	First author	Year	Country	Study design	Population	Sample size (n)	Surgical setting	Airway device (s)	Gastric tube / drainage strategy	Relevant outcomes reported	Key findings relevant to this review	Evidence tier
**Adachi_2024**	Adachi et al. Cureus. 2024;16:e67863.	Adachi	2024	Japan	Prospective manikin-based study with embedded practice survey	Manikin model (for GT insertion evaluation); 32 anesthesiologists (for practice survey)	Manikin: 4 SGA devices, multiple insertion attempts; Survey: 32 anesthesiologists	Not applicable (manikin-based evaluation)	i-gel; Ambu AuraGain; LMA ProSeal; LMA Supreme	Standardized gastric tube insertion through four second-generation SGAs in a manikin, plus survey of routine GT use	GT insertion time; ease of GT insertion; operator preference; survey of routine GT use	Supreme and AuraGain had the shortest gastric tube insertion times in the manikin model, and routine GT use was inconsistent in the accompanying survey.	Tier 3

Tier 3 comprised simulation, cadaveric, mechanistic, and practice-survey studies relevant to gastric tube placement through second-generation supraglottic airway devices. The included Tier 3 study used a dual-component design combining a manikin-based evaluation of gastric tube insertion performance with an embedded practice survey of anesthesia providers. Tier 3 evidence informed feasibility assessment, failure modes, and practice variation but was not used for effect estimation alongside Tier 1 and Tier 2 clinical evidence. Methodological appraisal for this study is provided in Supplementary Appendix 2B.

Abbreviations: GT, gastric tube; LMA, laryngeal mask airway; SGA, supraglottic airway.
